# Effectiveness of Direct Oral Anticoagulants in Obese Adults With Atrial Fibrillation: A Systematic Review of Systematic Reviews and Meta-Analysis

**DOI:** 10.3389/fcvm.2021.732828

**Published:** 2021-10-08

**Authors:** Fahad Shaikh, Rochelle Wynne, Ronald L. Castelino, Sally C. Inglis, Caleb Ferguson

**Affiliations:** ^1^Western Sydney Nursing and Midwifery Research Centre, Western Sydney University & Western Sydney Local Health District, Blacktown, NSW, Australia; ^2^School of Nursing and Midwifery, Deakin University, Geelong, VIC, Australia; ^3^Faculty of Medicine and Health, University of Sydney, Sydney, NSW, Australia; ^4^Pharmacy Department, Blacktown Hospital, Western Sydney Local Health District, Blacktown, NSW, Australia; ^5^Improving Palliative, Aged and Chronic Care Through Clinical Research and Translation (IMPACCT), University of Technology Sydney, Sydney, NSW, Australia

**Keywords:** atrial fibrillation, obesity, anticoagulant, direct oral anticoagulants, body mass index, pharmacology

## Abstract

**Background:** Atrial Fibrillation (AF) is the most common sustained cardiac arrhythmia. Obesity is an independent risk factor for AF. Anticoagulants have been strongly recommended by all international guidelines to prevent stroke. However, altered pathophysiology in obese adults may influence anticoagulant pharmacology. Direct oral anticoagulants (DOACs) in the context of obesity and AF have been examined in recent systematic reviews. Despite the similarities in included studies, their results and conclusions do not agree.

**Methods and Results:** The protocol for this review was registered with PROSPERO (CRD42020181510). Seven key electronic databases were searched using search terms such as “atrial fibrillation,” “obese,^*^” “overweight,” “novel oral anticoagulant,” “direct oral anticoagulant,” “DOAC,” “NOAC,” “apixaban,” dabigatran,” “rivaroxaban,” and “edoxaban” to locate published and unpublished studies. Only systematic reviews with meta-analyses that examined the effect of DOACs in overweight or obese adults with AF, published in the English language, were included. A total of 9,547 articles were initially retrieved. After removing the duplicates, title and abstract review and full-text review, five articles were included in the systematic review. From these only RCTs were included in the meta-analyses. There was disagreement within the published systematic reviews on DOACs in obesity. The results from our meta-analysis did not show any significant difference between all body mass index (BMI) groups for all outcomes at both 12 months and for the entire trial duration. Non-significant differences were seen among the different types of DOACs.

**Conclusion:** There was no difference between the BMI classes in any of the outcomes assessed. This may be due to the limited number of people in the trial that were in the obese class, especially obese class III. There is a need for large prospective trials to confirm which DOACs are safe and efficacious in the obese class III adults and at which dose.

## Introduction

Atrial Fibrillation (AF) is the most common sustained cardiac arrythmia. Major clinical sequela of AF includes systemic embolism, stroke, impaired cardiac function and heart failure (1, 2). Obesity is an independent risk factor for AF with underlying mechanisms that have a pathophysiological impact on AF (3–6). It is estimated that almost one in five cases of AF are attributed to obesity, to the extent that there is a 4 to 5% increase in AF risk for each incremental increase in body mass index (BMI) (7, 8).

The use of anticoagulants has been strongly recommended by all international guidelines, for AF patients that have a high risk of stroke (CHA_2_DS_2_-VASc score ≥ 2) (9–11). These guidelines recommend the use of direct oral anticoagulants (DOACs) rather than warfarin due to the significant association with higher rates of major bleeding, multiple food and drug interactions and the need for frequent monitoring (9, 10, 12–17). The altered pathophysiology in obese adults can influence the pharmacology of anticoagulants such as warfarin, thus requiring a higher dose and a longer time to reach therapeutic targets when compared to adults of normal weight (18). This may contribute to adverse events such as stroke and hospitalization because of anticoagulant under-dosing.

Despite the well-recognized cardiovascular consequences of obesity, there is a counterintuitive phenomenon known as the obesity paradox that has been hypothesized in some systematic reviews and meta-analyses (19, 20). In this phenomenon, overweight and mildly obese (BMI < 35 kg/m^2^) participants that were in the DOAC group, appear to have lower all-cause mortality in studies with longer-term follow up. Despite this finding, several studies have critiqued the assertion based on the potential for spurious associations with rhythm control strategies, unreported confounders, limitations of anthropometric markers such as BMI in assessing adiposity and selection bias in observational or cohort studies (6, 8, 21–23).

DOACs have been the focus of attention in several systematic reviews (19, 20, 24–27), exploring their use in obesity. Recommendations from these studies appear to be conflicting. The effect of the obesity paradox in the context of AF, or robust data comparing the effectiveness of DOACs with warfarin, remain elusive. Product information documents supporting DOAC use indicate that dose adjustment is not required for any of the DOACs (28–30). However, in the clinical trials conducted to inform the product information documents, such as ARISTOTLE, RE-LY and ROCKET-AF (31–33), weight classes were not equally distributed. For example, most of the participants enrolled in the dabigatran clinical trials (up to 80%) were between 50 and 100 kg (29). Participants in the ARISTOTLE trial (34) that were >140 kg were under-represented comprising only 1.4% of the entire trial population. Both the International Society on Thrombosis and Haemostasis (ISTH) and the European Society of Cardiology (ESC) Working Group on Thrombosis have questioned the use of DOACs in morbidly obese adults (i.e., BMI ≥ 40 kg/m^2^), due to the extremely limited or absent clinical data (35). The ISTH have suggested that DOACs should not be used in BMI of >40 kg/m^2^ or >120 kg (36). Although guidance from ISTH provides an alternative option for DOAC use in obesity, there have been no original research studies that have examined its effectiveness in the obese population or compared the effectiveness of DOACs exclusively according to BMI category. Given the high-risk clinical consequences of anticoagulants, a better understanding of the safety and efficacy of DOACs in obese adults with AF is warranted. The aim of this systematic review is to evaluate the current evidence on the safety and effectiveness of direct oral anticoagulants (DOACs) in obese adults with AF.

## Methods

This systematic review was conducted in accordance with gold-standard systematic review and meta-analysis methodology informed by the Cochrane Collaboration and the Joanna Briggs Institute (JBI) methodology for systematic reviews of effectiveness evidence (37, 38). The review protocol has been registered with the PROSPERO register (CRD42020181510).

### Search Strategy

The search strategy used key search terms such as “atrial fibrillation,” “obese,^*^” “overweight,” “novel oral anticoagulant,” “direct oral anticoagulant,” “DOAC,” “NOAC,” “apixaban,” dabigatran,” “rivaroxaban,” and “edoxaban” (see Supplementary Table 5 for full search strategy). It was designed to locate published and unpublished studies. Text words contained in the titles and abstracts of relevant articles and the index terms used to describe the articles were used to develop a full search strategy. The reference lists of all studies selected for critical appraisal were screened for additional studies that were then included in this study.

### Inclusion and Exclusion Criteria

Only systematic reviews with meta-analyses that examined the effect of DOAC in overweight or obese adults with AF, published in the English language, were included. Studies were excluded if they were related to interventional studies (for example, cardioversion, catheter ablation and gastric bypass) and not a systematic review or a systematic review with meta-analysis (for example, *post-hoc* analysis, abstracts, conference proceedings, review paper, observational or retrospective cohort studies, editorials, and commentaries) (see Supplementary Table 1). Any non-RCT such as *post-hoc* analysis of a RCT, observational studies included in the systematic reviews and/or meta-analysis were excluded in this meta-analysis (see Supplementary Table 2). Studies that were published before 2005 were also excluded as prior to this time no DOAC trials had commenced.

### Outcomes

Primary outcomes assessed were stroke (ischemic or hemorrhagic) or systemic or pulmonary embolism. Secondary outcomes assessed included all-cause mortality, transient ischemic attack, myocardial infarction, major bleed, all-cause hospitalization, and cardiovascular mortality. Outcomes were assessed at 12 months and for the entire trial duration.

### Data Sources

Seven key electronic databases were searched including Medline, CINAHL, Scopus, Web of Science, Cochrane Database of Systematic Reviews, Johanna Briggs Institute and Embase. Clinical trial registries were checked to ensure all relevant trials were identified. The fidelity of the search strategy was tested and confirmed by two investigators (FS, CF) who independently implemented the search and compared findings from each database. Search findings were downloaded into EndNote X9.3 (39) citation management software.

### Study Selection

**Figure 1:**
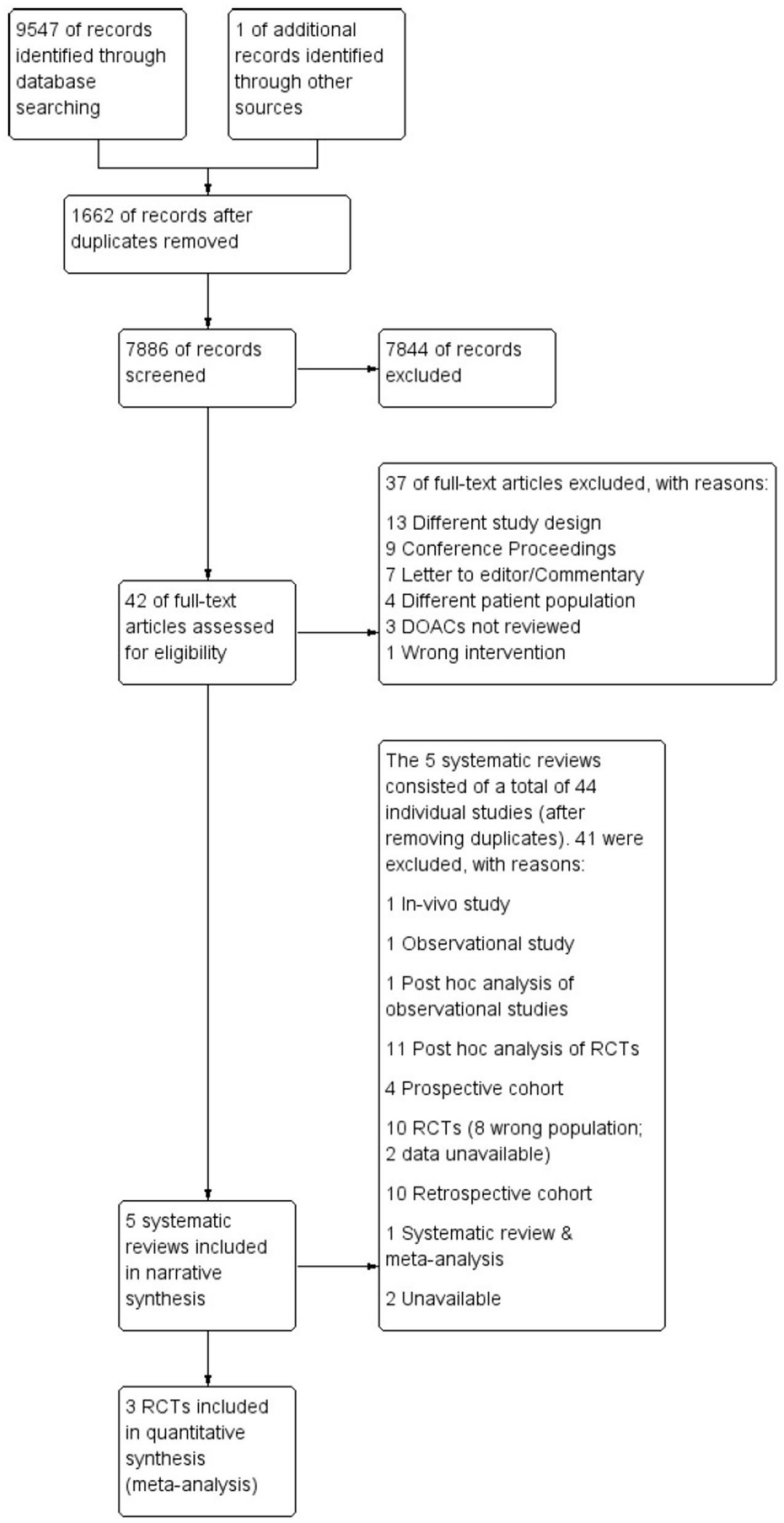
PRISMA flowchart.

Following the search, all identified citations were uploaded into Covidence systematic review software (40) and duplicates removed. Titles and abstracts were screened for assessment against review inclusion and exclusion criteria. Full text of selected citations was assessed in detail against the inclusion and exclusion criteria. The entire screening process was undertaken by two investigators (FS, CF) at each stage of the study selection process and disagreements were resolved through consensus discussion with a third arbitrary investigator (RW). The results of the search are reported in full and presented in a Preferred Reporting Items for Systematic Reviews and Meta-analyses (PRISMA) flow diagram (41) as shown in Figure 1.

### Assessment of Methodological Quality

The quality of eligible studies was critically appraised by two investigators using a standardized critical appraisal instrument: The Assessment of Multiple Systematic Reviews (AMSTAR-2) tool (42). Any disagreements that arose were resolved through discussion, or review by a third investigator. The results of the critical appraisal are reported in narrative form in Table 1. Risk of bias was assessed using the ROBIS tool for risk of bias in systematic reviews (76).

**Table 1:** Study characteristics.

**Study name**	**Zhou et al. (20)**	**Proietti et al. (19)**	**Boonyawat et al. (27)**	**Malik et al. (25)**	**Kido et al. (43)**
Study design	Systematic Review and Meta-Analysis	Systematic Review and Meta-Analysis	Systematic Review and Meta-Analysis	Meta-Analysis	Meta-Analysis
Study population	AF patients with anticoagulants	AF patients with or without anticoagulants	AF and VTE patients	AF patients with anticoagulants	Morbidly obese AF patients with anticoagulants
Aim	To explore if there is an obesity paradox in anticoagulated AF patients, and compare the treatment effects between DOACs and warfarin in AF patients across BMI categories.	(1) To provide a comprehensive report of all available evidence on the relationship between overweight and obesity in AF patients	To investigate the association of body weight and patient-important outcomes in patients treated with DOACs or warfarin, and to demonstrate the fixed-dose effect of DOACs	To investigate the clinical consequences of the use of DOACs in patients with NVAF within various BMI categories.	To compare DOACs with warfarin in morbidly obese patients with AF and to optimize an anticoagulation therapy in the population.
		(2) To perform comparative analysis of observational studies subgroup analyses from RCTs			
		(3) To conduct a meta-analysis of available data on the relationship of BMI to stroke/systemic embolic event and major bleeding in the phase III DOAC trials of stroke prevention in AF			
Interventions and comparisons	DOACs vs. Warfarin across the BMI categories	DOACs vs. Warfarin across the BMI categories	DOACs vs. Warfarin across the BMI categories	DOACs vs. Warfarin across the BMI categories	DOAC vs. Warfarin
Inclusion	(1) Phase III RCTs, *post-hoc* analyses of RCTs, or observational cohorts (prospective or retrospective)	(i) Both RCTs and observational cohort studies focusing on patients with established AF.	Subgroups of phase III RCTs investigating DOACs, including dabigatran, rivaroxaban, apixaban and edoxaban, for the prevention of stroke and systemic embolism in AF and in acute VTE treatment, or sub-studies or subgroup analysis of the phase III RCTs.	RCTs that had the comparative data of DOACs or warfarin treatment according to the different weight categories, including underweight, overweight, obese, or any subcategories based on BM	Included patients that are aged > 18 years old with BMI > 40 kg/m^2^ or weight > 120 kg receiving warfarin, apixaban, dabigatran, edoxaban, or rivaroxaban who are diagnosed as AF
	(2) Reported the impact of BMI on any outcome (i.e., SSE, all-cause death, and major bleeding) in NVAF patients with DOACs or warfarin	(ii) Specific data on BMI and BMI categories.			
	(3) Reported BMI as a categorical or continuous variable.	iii) Studies reporting data on long-term follow-up observations.			
Exclusion	(1) Included AF patients with interventions (e.g., ablation, cardioversion, or coronary interventions) or with other coexisting diseases (e.g., acute coronary syndrome, HF, carotid artery disease, and cancer)	(i) Conference abstracts, letters, comments, case reports, and editorials.	DOACs for primary prevention of VTE in orthopedic surgery and medically ill patients, extended treatment of VTE or other indications (acute coronary syndrome, atrial thrombus, perioperative management, and antiphospholipid syndrome)	N/A	Included mechanical heart valve recipients, pregnant or dialysis patients. Non-English articles, case series, case-control studies and meta-analyses were excluded. Meeting abstracts
	(2) Were certain publication types (e.g., reviews, comments, editorials, letters, conference abstracts, and animal studies)	ii) Studies not published in English			
Outcomes	SSE, all-cause death, major bleeding	Meta-analysis: SSE & major bleeding; descriptive analysis: All AF related outcomes e.g., CV death, all-cause death, SEE, major bleeding, MI etc.	Thromboembolic outcomes including stroke and/or systemic embolism in AF studies and symptomatic recurrent VTE or VTE-related death in VTE studies were recorded.	Efficacy: events of SSE Safety: major bleeding and all-cause mortality.	Primary efficacy outcome is the composite outcome of stroke or SE and primary safety outcome is the major bleeding event rate.
			Bleeding outcomes, including major bleeding as defined by the ISTH (9) and/or clinically relevant non-major bleeding (CRNMB)		
Number of databases searched	PubMed (*n =* 66), Embase (*n =* 334)	PubMed (*n =* 231), Scopus (*n =* 256)	PubMed (*n =* 212), Medline (*n =* 2,614), Embase (*n =* 3,511), Other (*n =* 250)	PubMed, Cochrane library, Embase	Medline, Embase, Google Scholar, Web of Science and Cochrane Library
Included Studies	9 studies:	13 studies	14 studies	7 studies	5 studies
	RCT	For narrative analysis:	RCT	RCT	*Post-hoc* analysis of RCT
	Connolly et al. (32) (RE-LY)	*Post-hoc* analysis of RCT:	Schulman et al. (44) (RECOVER II), Schulman et al. (45) (RECOVER I), Bauersachs et al. (46) (EINSTEIN DVT), Buller et al. (47) (EINSTEIN PE), Agnelli et al. (48) (AMPLIFY), Buller et al. (49) (Hokusai-VTE), Connolly et al. (32) (RE-LY), Patel et al. (33) (ROCKET AF),	Connolly et al. (32) (RE-LY), Patel et al. (33) (ROCKET AF)	Hohnloser et al. (34) (ARISTOTLE)
	*Post hoc* analysis of RCT:	Ardestani et al. (50) (AFFIRM), Badheka et al. (51) (AFFIRM), Senoo et al. (52) (AMADEUS), Proietti et al. (19) (SPORTIF III and V), Sandhu et al. (53) (ARISTOTLE)	Granger et al. (31) (ARISTOTLE), Giugliano et al. (54) (ENGAGE AF-TIMI 48), Connolly et al. (55) (AVERROES)	*Post hoc* analysis of RCT	Retrospective cohort
	Sandhu et al. (53) (ARISTOTLE), Boriani et al. (56) (ENGAGE AF-TIMI 48), Proietti et al. (19) (SPORTIF III and V), Balla et al. (57) (ROCKET AF), Piccini et al. (58) (ROCKET AF), Hohnloser et al. (34) (ARISTOTLE)	Prospective cohort:	*Post-hoc* analysis of RCT	Sandhu et al. (59) (ARISTOTLE – Poster), Boriani et al. (56) (ENGAGE AF-TIMI 48)	Kushnir et al. (60), (61), Perales et al. (62), Peterson et al. (63)
	Retrospective cohort:	Overvad et al. (64) (Danish Diet, Cancer and Health study), Wang et al. (65) (Chinese ED admissions), Bunch et al. (66)	Eikelboom et al. (67) (RE-LY)	Balla et al. (57) (ROCKET AF), Hohnloser et al. (34) (ARISTOTLE)	
		(LDS Hospital or Intermountain Medical Center)			
	(68, 69)	Observational study:	Sandhu et al. (59) (ARISTOTLE—Poster)	Systematic review & Meta-analysis	
		Yanagisawa et al. (70) (Nagoya University Hospital)	Unknown	Proietti et al. (19)	
		Retrospective cohort	Prins et al. (71)		
		Wang et al. (72) (Chinese PLA General Hospital), Kwon et al. (73) (ARIC and CHS study), Pandey et al. (74) (ORBIT-AF registry)			
		*Post-hoc* analysis of observational data:			
		Inoue et al. (75) (J-RHYTHM Registry)			
		For Meta-analysis: Connolly et al. (32) (RE-LY), Patel et al. (33) (ROCKET AF), Sandhu et al. (53) (*Post hoc* of ARISTOTLE)	
Types of DOACs	Rivaroxaban, dabigatran, apixaban, edoxaban	Rivaroxaban, dabigatran, apixaban	Rivaroxaban, dabigatran, apixaban, edoxaban	Rivaroxaban, dabigatran, apixaban, edoxaban	Apixaban, rivaroxaban
BMI Categories	Underweight, normal weight, overweight, obese classes	Normal weight, overweight, obese	High weight, underweight, normal weight, obese	Low bodyweight, normal weight, overweight, obese classes	BMI > 40 kg/m^2^ or weight > 120 kg
Conclusion	DOACs have better efficacy and safety profiles than warfarin in underweight, normal weight and overweight patients, but are not inferior to warfarin in obese patients. There may be an obesity paradox in anticoagulated patients with AF	There may be an obesity paradox in AF patients, particularly for all-cause and cardiovascular death outcomes. RCT trials showed overweight and obese patients reporting a lower risk for SSE event. For major bleeding, only obese patients were at lower risk compared with normal weight patients. However, observational cohorts did not show this relationship.	Patients with low body weight had a paradoxical increase in the risk of thromboembolism compared with non-low body weight patients. The subgroup of AF patients with a high body weight had a favorable thromboembolic outcome compared with AF patients with a non-high body weight. Dose adjustment of DOACs, outside that recommended in the package insert, is unlikely to improve safety or efficacy.	For NVAF patients with extremes of weight, DOACs appear to be similarly effective and safer than warfarin for reduction of SSE. With an increasing BMI, the meta-regression analysis confirms less substantial benefit with DOACs compared with warfarin, suggesting that weight-based dosage adjustment with drug monitoring may be warranted in severely obese patients	DOAC use was not associated with the higher event rate of stroke or SE compared to warfarin therapy in morbidly obese patients with AF but a DOAC was associated with significantly lower rate of major bleeding compared to warfarin.
					A RCT comparing a DOAC with warfarin is needed to confirm our meta-analysis results, although it may not be feasible.
AMSTAR Score	Low quality	Low quality	Low quality	Critically low quality	Moderate quality
ROBIS	Low	Unclear	Low	Unclear	High

*NVAF, Non-valvular Atrial Fibrillation; MI, Myocardial Infarction; SE, Systemic Embolism*.

### Data Extraction

Data was extracted from studies included in the review using a standardized data extraction tool. The data extracted included specific details about the study population, methods, interventions, and outcomes of significance to the review objective. Any disagreements that arose between the reviewers (FS, CF) were resolved through discussion, or with a third investigator (RW). Authors of all the five DOAC trials that met our inclusion criteria for the meta-analysis were contacted by email to request the data as the published data did not enable stratification by BMI. Authors of three studies (RE-LY, AVERROES, and ENGAGE AF-TIMI 48) agreed to share data for the purposes of a meta-analysis. Two of the trials, ARISTOTLE, and ROCKET-AF, did not provide data stratified by BMI and were excluded from the meta-analysis. Data was analyzed using the intention to treat cohort in all trials to minimize any risk of bias.

### Data Synthesis

Meta-analysis was performed using only RCTs from eligible systematic reviews to minimize risk of bias that can arise from other study designs. Data from only the DOAC group in the trials were pooled for statistical meta-analysis using RevMan 5.3 (77). Effect sizes were expressed as odds ratios (for dichotomous data) with 95% confidence intervals. Heterogeneity was assessed statistically using the standard chi-square and *I*^2^ tests. Statistical analyses were performed using the DerSimonian and Laird Method for random effects meta-analysis.

### Deviation From Protocol

There have been three deviations from the registered protocol on PROSPERO. The first was that this paper also includes further analysis of the different BMI groups rather than the two groups noted in the registered protocol. The second major deviation was that a summary of findings is not provided as the risk of bias was only completed for systematic reviews, not primary studies, as these have previously been assessed for risk of bias when included in the original systematic reviews. The last deviation is that publication bias assessment was also excluded as it was not required, as per the Cochrane Handbook (38), due to the number and type of studies included in this systematic review.

## Results

### Search Results

As illustrated in Figure 1, a total of 9,547 articles were initially retrieved. After removing the duplicates (*n* = 1,662), 7,844 articles were excluded after title and abstract review, leaving 42 articles for full-text review. A further 37 articles were excluded for reasons listed in Supplementary Table 1, leaving five articles that met inclusion criteria. The five systematic reviews comprised 40 individual original studies after removing duplicates; 11 RCTs, 11 *post-hoc* analyses of RCTs, nine retrospective cohort studies, three prospective studies, one observational study, one *post-hoc* analysis of observational data, a systematic review and meta-analysis and a conference abstract (see Supplementary Table 2). As stated in the methods, only RCTs were included in meta-analyses. Six RCTs focused on Venous Thromboembolism (VTE) and Pulmonary Embolism (PE), hence were excluded from the meta-analysis. Of the remaining five trials that focused on AF, only three of the five authors of the trials agreed to share data for the meta-analysis. Thus, two of the trials, ARISTOTLE, and ROCKET-AF, were excluded from the meta-analysis and only the RE-LY, AVERROES and ENGAGE AF-TIMI 48 trials were included.

### Description of Included Studies in Narrative Synthesis

Table 1 provides a summary of the characteristics of the included reviews. In brief, all studies except for Kido et al. (43) evaluated the effect of DOACs vs. Warfarin across different weight groups. Kido et al. (43) only evaluated the effect of DOACs vs. Warfarin in obese groups (BMI > 40 or > 120 kg). Similarly, all studies evaluated the effected of DOACs vs. Warfarin in AF, apart from Boonyawat et al. (27) who also included VTE patients. Stroke or systemic embolism (SSE) and major bleeding were the primary efficacy and safety outcomes in all studies, however, some studies also reported outcomes such as all-cause death and cardiovascular death. Proeitti et al. (19) and Boonyawat et al. (27) provided the most comprehensive systematic reviews based on the number and type of included studies.

Despite the comprehensiveness with regards to the quantity and similarity of the included studies, the five systematic reviews did not have complete agreement in their results and conclusion, nor was the comprehensiveness reflected in the quality and risk of bias assessment, as discussed in the next section. Zhu et al. (26) and Proietti et al. (19) concluded that “… there appears to be an obesity paradox in obese adults with atrial fibrillation” and a superior efficacy and safety profile for DOACs in overweight and obese adults. Conclusions from Boonyawat et al. (27) were similar to the aforementioned studies but alluded to variability in baseline characteristics influencing outcome. Malik et al. (25) and Kido et al. (43) reached similar conclusions with no significant difference between DOACs and warfarin with regards to efficacy, however they reported better safety outcomes for DOACs compared to warfarin. Both reviews recommended further trials comparing DOACs to Warfarin to confirm their findings, in addition to suggesting the need for weight-based dosage adjustment with drug monitoring in such trials.

### Methodological Quality and Risk of Bias Assessment

Quality assessment and risk of bias were undertaken using the AMSTAR-2^©^ and ROBIS^©^ tools (42, 76). Table 2 provides a summary of the risk of bias assessment. Three of the five systematic reviews were assessed as low quality. Zhou et al. (20) and Boonyawat et al. (27) had low risk of bias due to the thoroughness in their methodology and the quantity/quality of included studies. Zhou et al. (20) did not provide any justification for combining different study designs into the same analysis or why they had excluded some trials in the grouped analysis but included them in individual analysis. The review authors stated that they had extracted “underweight data from Hohnloser et al. (34) and overweight/obese data from Sandhu et al. (53).” However, these original studies used different definitions of weight groups, that is, Hohnloser et al. (34) stratified using actual weight and Sandhu et al. (53) used BMI. Boonyawat et al. (27) had used the Mantel-Haenszel method instead of the Laird Method to analyze the data which they determined to be of random effects and had defined high body weight as a minimum of 100 kg, which may have lacked clinical sensitivity.

**Table 2:** Risk of bias using ROBIS tool.

**Review**	**Phase 2**				**Phase 3**
	**1. Study eligibility criteria**	**2. Identification and selection of studies**	**3. Data collection and study appraisal**	**4. Synthesis and findings**	**Risk of bias in the review**
Zhou et al. (20)	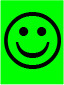	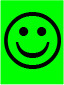	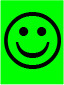	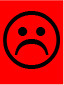	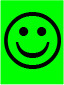
Proietti et al. (19)	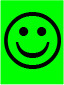	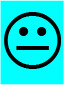	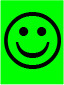	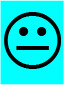	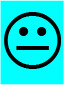
Boonyawat et al. (27)	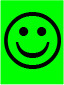	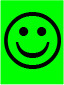	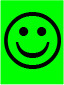	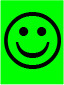	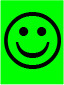
Malik et al. (25)	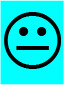	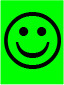	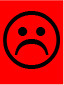	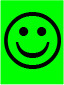	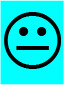
Kido et al. (43)	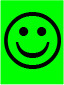	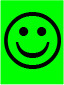	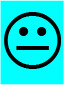	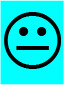	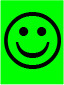

Proietti et al. (19) was also assessed as low quality but had unclear risk of bias, due to several issues. Firstly, the authors mentioned that they had used *I*^2^ to determine if there was heterogeneity in the trial. However, given there were different doses and drugs used across the different trials, heterogeneity would have been intrinsic. Fixed method modeling instead of random with the Laird Method was used for their analysis which is not consistent with heterogeneity. Secondly, the event numbers that the authors presented in their forest plots did not correspond to the event numbers we received from the trial authors. The authors did not provide a justification for combining different study designs into the same analysis; observational studies were included. Risk of bias was only completed for the studies included in the meta-analysis, without any justification for excluding the studies included in the narrative synthesis. Lastly, the authors mentioned they also relied on data from regulatory submissions for dabigatran and rivaroxaban; however, they did not specify which trial was included as part of their data extraction.

Malik et al. (25) was assessed as critically low quality with an unclear risk of bias. This was predominantly due to the lack of clarity and risk of bias assessment, limited comprehensiveness in their literature search and justification behind its exclusion of articles. Additionally, in the methods, the authors stated that the RR would be reported, but ORs were reported throughout, with no justification for change in reporting measure.

Although the quality assessment of Kido et al. (43) was the highest of all the included reviews, a high risk of bias was revealed. This was due to the unjustified exclusion of all the DOAC trials and the *post-hoc* analysis of the RCTs, as well as other relevant key studies. Along with Zhou et al. (20) and Proietti et al. (19), Kido et al. (43) also used the Mantel-Haenszel method instead of the Laird Method to analyze the data, which they determined to be of random effects. There was no justification for combining different study designs into the same analysis and the data extracted from Hohnloser et al. (34) may not be accurate; in **Figure 3**, the DOAC event states 13/480, however, in the paper by Hohnloser et al. (34) the event rate is 13 per 100 per year. Kido et al. (43) had reported this number over a 4-year period.

### Meta-Analysis of Data From DOAC Trials

Data were obtained by contacting the study authors of all five DOAC trials (31–33, 54, 55) as the data from the *post-hoc* analysis of the RCTs did not have adequate information to conduct a meta-analysis for our intended subgroup analysis. Only the ENGAGE AF-TIMI 48 trial reported transient ischemic attack (TIA) and only two trials, ENGAGE AF-TIMI 48 and RE-LY, reported all-cause hospitalization.

**Figure 2:**
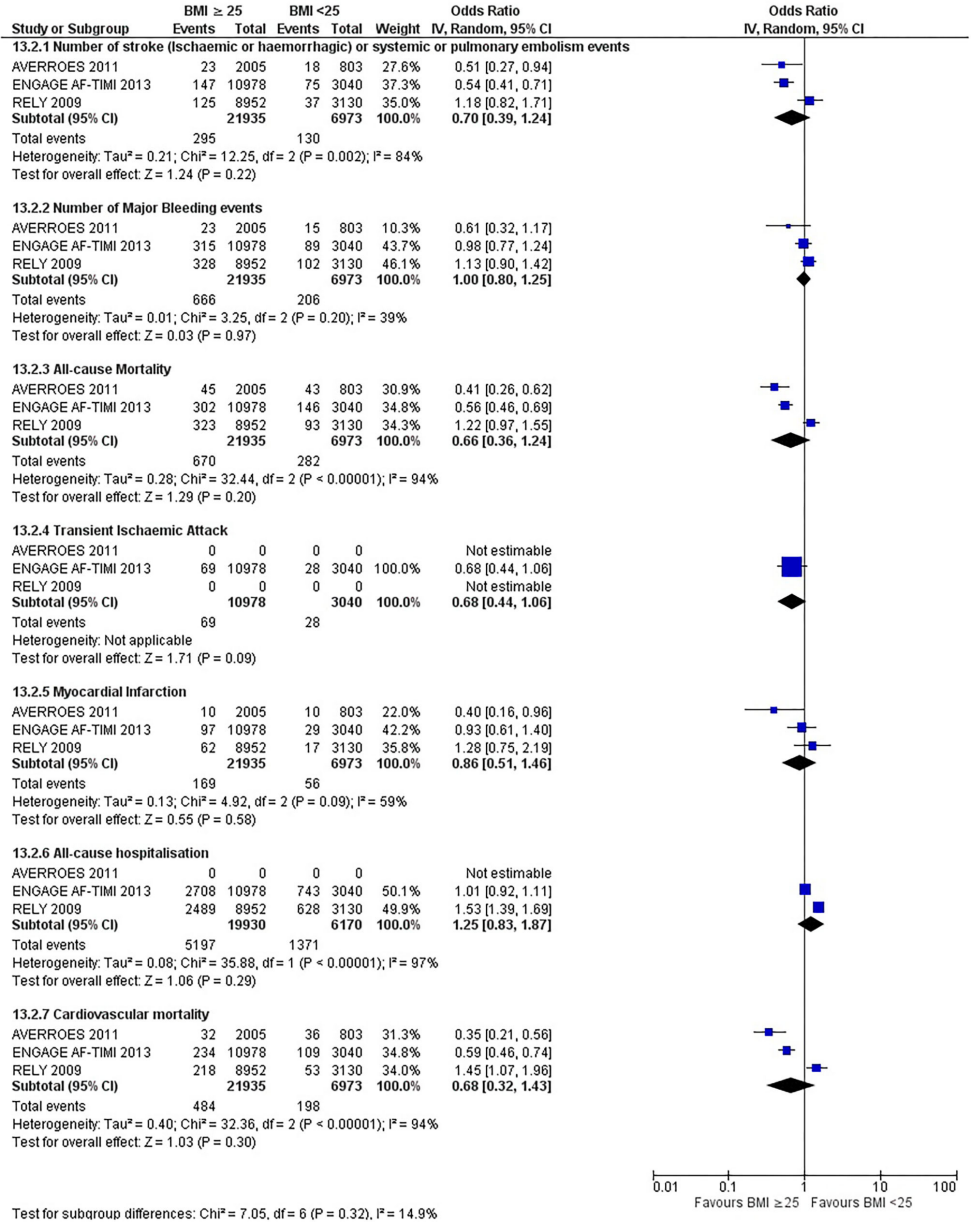
Forest plot of comparison: BMI ≥ 25 vs. BMI <25 at 12 months.

**Figure 3:**
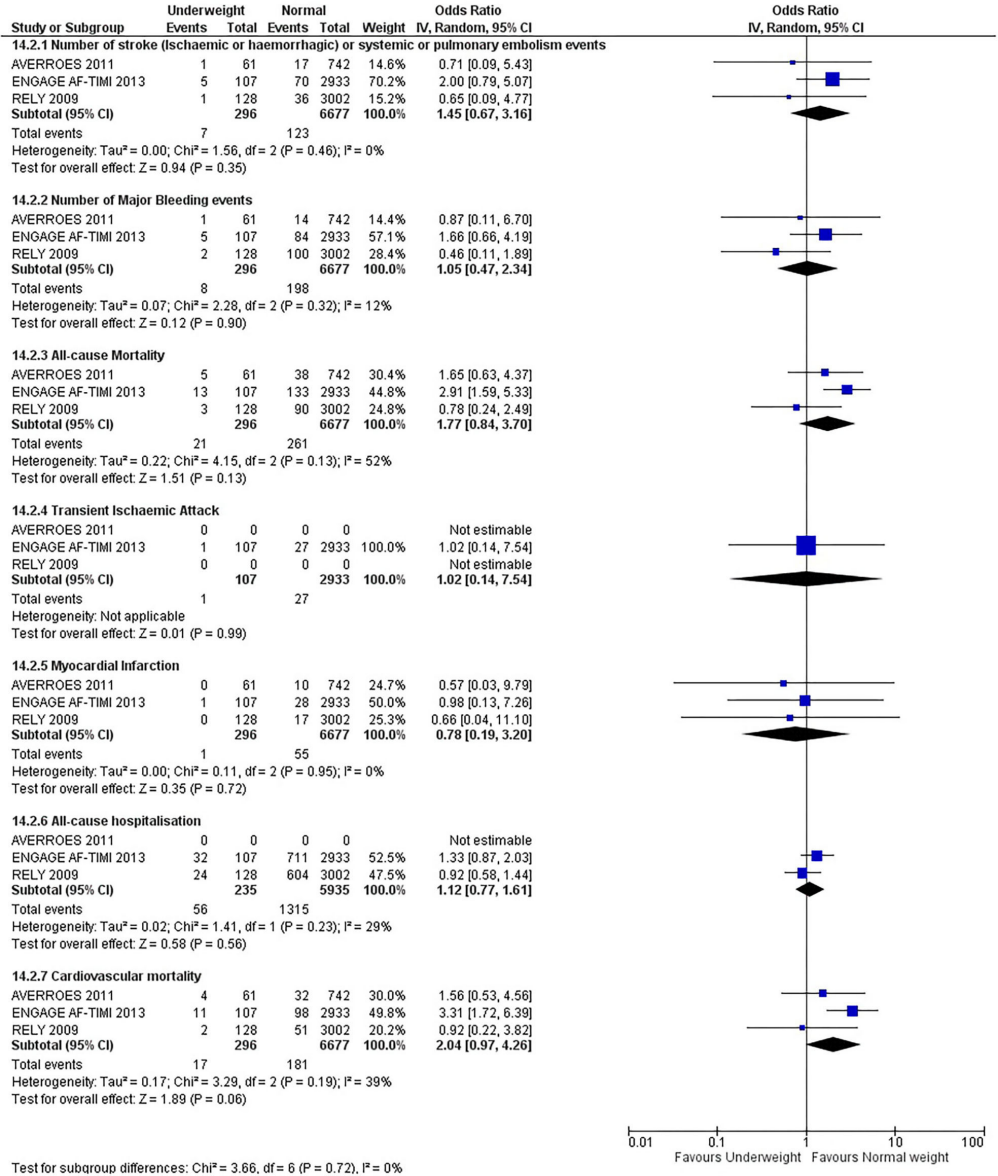
Forest plot of comparison: Normal vs. Underweight at 12 months.

**Figure 4:**
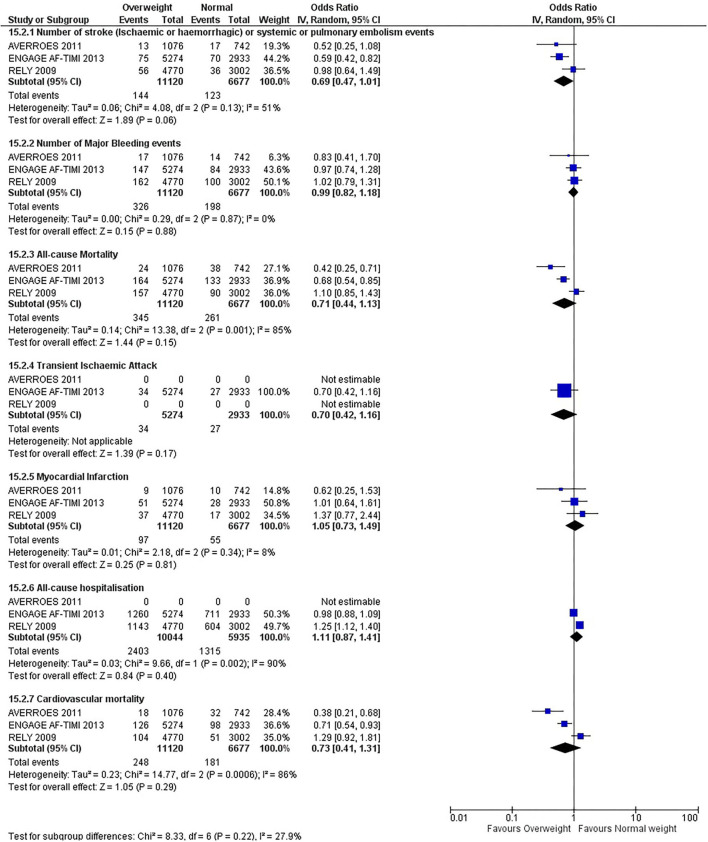
Forest plot of comparison: Normal vs. Overweight at 12 months.

**Figure 5:**
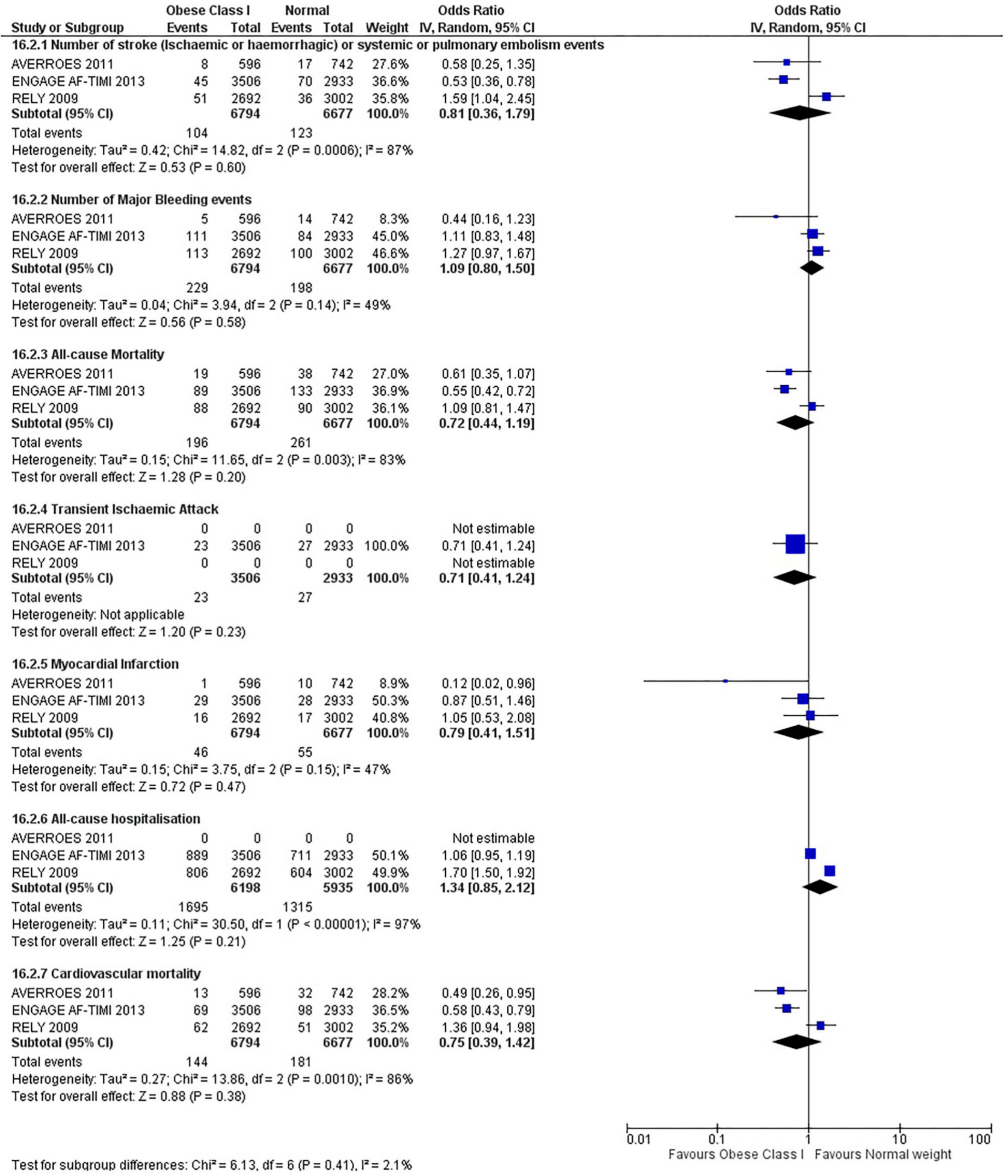
Forest plot of comparison: Normal vs. Obese class I at 12 months.

**Figure 6:**
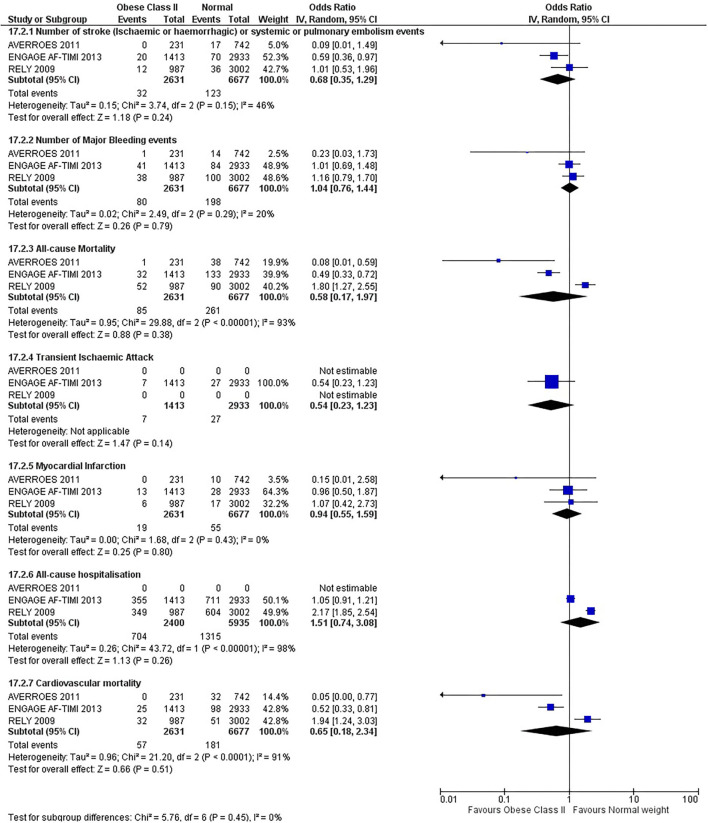
Forest plot of comparison: Normal vs. Obese class II at 12 months.

**Figure 7:**
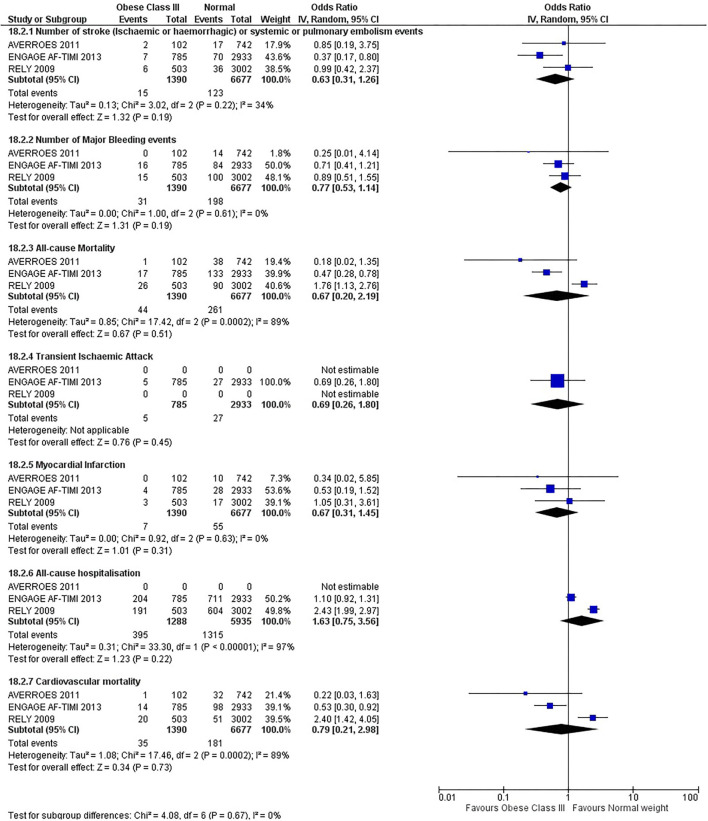
Forest plot of comparison: Normal vs. Obese class III at 12 months.

Our initial analysis had grouped the populations as either overweight/obese or normal/underweight. There was no significant difference between the two groups for any outcomes at 12 months (see Figures 2–7). Similarly, there was no significant difference between the different BMI groups when compared with normal BMI. However, we did notice a common trend across all analyses; there were differences in the results from the individual trials, suggesting there might be differences in the individual agents among the different weight groups. The primary efficacy outcome of stroke and primary safety outcome of major bleeding did not show any significant difference between any BMI groups.

There was, however, a difference between dabigatran (RE-LY 2009), apixaban (AVERROES 2011) and edoxaban (ENGAGE AF-TIMI 48 2013), where overall, dabigatran was favorable in the normal weight group when compared to overweight and obese classes for all-cause mortality (OR, 1.80; 95% CI, 1.27–2.55 [obese class II vs. normal]; OR, 1.76; 95% CI, 1.13–2.76 [obese class III vs. normal]), all-cause hospitalization (OR, 1.25; 95% CI, 1.12–1.40 [overweight vs. normal]; OR, 1.70; 95% CI, 1.50–1.92 [obese class I vs. normal]) OR, 2.17; 95% CI, 1.8–2.54 [obese class II vs. normal]) OR, 2.43; 95% CI, 1.99–2.97 [obese class III vs. normal]) and cardiovascular mortality (OR, 1.94; 95% CI, 1.24–3.03 [obese class II vs. normal]; OR, 2.40; 95% CI, 1.42–4.05 [obese class III vs. normal]). Dabigatran was also favorable in the BMI ≤ 25 group for all-cause hospitalization (OR, 1.53; 95% CI, 1.39, 1.69) and cardiovascular mortality (OR, 1.45; 95% CI, 1.07, 1.96) outcomes in the BMI ≥ 25 vs. BMI ≤ 25 comparison. Furthermore, data from the entire trial suggested that dabigatran was favorable in the normal group when compared to the obese class III for stroke (OR, 2.00; 95% CI, 1.23–3.27) and major bleeding (OR, 1.59; 95% CI, 1.11–2.26).

In contrast, apixaban was favorable in the overweight (OR, 0.42; 95% CI, 0.25, 0.71) and obese class II (OR, 0.08; 95% CI, 0.01, 0.59) group for all-cause mortality, and among the overweight (OR, 0.38; 95% CI, 0.21–0.68), obese class I (OR, 0.49; 95% CI, 0.26–0.95) and obese class II (OR, 0.05; 95% CI, 0.00–0.77) groups, for cardiovascular mortality in the overweight, obese class I and obese class II vs. normal weight comparisons. In the BMI ≥ 25 vs. BMI ≤ 25 comparison, apixaban was favorable in the BMI ≥ 25 group for stroke (OR, 0.51; 95% CI, 0.27–0.94), all-cause mortality (OR, 0.41; 95% CI, 0.26–0.62) and cardiovascular mortality (OR, 0.35; 95% CI, 0.21–0.56) outcomes.

Similarly, edoxaban (ENGAGE AF-TIMI 48) was favorable in the overweight and all obese classes for stroke (OR, 0.59; 95% CI, 0.42–0.82 [overweight vs. normal]; OR, 0.53; 95% CI, 0.36–0.78 [obese class I vs. normal]; OR, 0.59; 95% CI, 0.36–0.97 [obese class II vs. normal]; OR, 0.37; 95% CI, 0.17–0.80 [obese class III vs. normal]), all-cause mortality (OR, 0.68; 95% CI, 0.54–0.85 [overweight vs. normal]; OR, 0.55; 95% CI, 0.42–0.72 [obese class I vs. normal]; OR, 0.49; 95% CI, 0.33–0.72 [obese class II vs. normal]; OR, 0.47; 95% CI, 0.28–0.78 [obese class III vs. normal]) and cardiovascular mortality (OR, 0.71; 95% CI, 0.54–0.93 [overweight vs. normal]; OR, 0.58; 95% CI, 0.43–0.79 [obese class I vs. normal]; OR, 0.52; 95% CI, 0.33–0.81 [obese class II vs. normal]; OR, 0.53; 95% CI, 0.30–0.92 [obese class III vs. normal]) in the overweight and obese vs. normal comparisons. In the BMI ≥ 25 vs. BMI ≤ 25 comparison, edoxaban was favorable in the BMI ≥ 25 group for stroke (OR, 0.54; 95% CI, 0.41–0.71), all-cause mortality (OR, 0.56; 95% CI, 0.46–0.69) and cardiovascular mortality (OR, 0.59; 95% CI, 0.46–0.74) outcomes.

The analysis was repeated using data collected for the entire trial duration to explore differences resulting from a potential lack of power in data from 12 months (see Supplementary Figures 1–6). Our analysis revealed results similar to those reported at 12 months, where no significant difference was found between any of the subgroups. Additionally, we also noticed similar trends to that at 12 months, where there some difference with regards to the favorable subgroups when comparing the different DOACs. In summary, dabigatran was overall more favorable in the normal BMI group when compared to the different obese classes. This was in contrast with apixaban and edoxaban, where overall they were more favorable in the overweight/obese classes when compared to the normal BMI group. Supplementary Table 4 provides a summary of the differences between DOACs at both time points.

## Discussion

There appears to be disagreement within the published systematic reviews on the use of DOACs in obese adults with AF. Data extraction inconsistencies and appropriateness of the statistical methods used in the analysis of the trials warrant further validation of the findings of the studies.

This meta-analysis did not show any significant difference between all BMI groups at 12 months or for the entire trial duration for all outcomes. The results do not indicate the presence of the obesity paradox for DOACs overall, although individual superiority may exist, which contrasts with the findings of Zhou et al. (20) and Proietti et al. (19).

We did, however, notice differences and trends, although not significant, among the different types of DOACs. Dabigatran was favorable overall in the normal weight group compared to overweight and obese classes predominately for stroke, major bleeding, all-cause mortality, all-cause hospitalization, and cardiovascular mortality. This contrasts with the results for apixaban and edoxaban, where these drugs were overall favorable in the overweight/obese classes. A similar observation was also found in a retrospective cohort study and a recent review of literature (61, 78).

Although our findings are not statistically significant or conclusive, the consistent trend across most of the analysis of the BMI groups, and new data from the literature, suggests there may be differences in the individual agents among the different weight groups. However, this would need to be further evaluated by future prospective trials and meta-analysis to contrast DOACs and evaluate the effect of dose differences of specific DOACs in obese adults.

While the original systematic reviews suggest the presence of an obesity paradox, they also point toward several underlying reasons for this. These include changes in baseline characteristics, that is, BMI, and dominance in data from subgroup analysis of RCTs, compared to data from observational studies after statistical adjustments for confounding factors (19, 27).

Over recent years, there have been numerous studies that have examined and alluded to the existence of the obesity paradox in multiple conditions such heart failure, diabetes, and now AF (22, 79). However, many of these studies fail to address or explore the possible reasons behind the “illusion” of the obesity paradox, despite the well-known consequences of obesity, which ironically is a risk factor of cardiovascular disease.

These findings are often found in *post-hoc* analysis of RCTs, where the authors also acknowledge the lack of recorded follow-up data regarding weight change or nutritional behavior as a limitation (19, 27, 79). This illuminates the importance of changes in baseline characteristics and lack of recording of any physical and nutritional changes that may occur in participants in RCTs. Lavie et al. (8) have also argued for the involvement of other confounding factors such as age and management disparity within the BMI groups, where higher BMI groups were significantly younger and had greater use of rhythm, rate and anticoagulant interventions compared to normal BMI groups (8).

Furthermore, due to the well-known complications and negative effects of obesity, over 50% of physicians advise patients to lose weight and to maintain a healthy diet (80). Studies have shown that physical activity can modify anticoagulation (warfarin) response by affecting blood fluidity (81–83). It has also been hypothesized that the presence of the obesity paradox is largely related to differences in cardiorespiratory fitness levels (8).

Although RCTs are considered the highest level of evidence for experimental studies, the lack of recording of any changes in baseline characteristics at follow up can influence the results, especially when *post-hoc* analyses are undertaken. Additionally, due to the strict inclusion and exclusion criteria many participants are not able to be included in the trial (84, 85). Studies have shown that up to 50–75% of patients that will end up being prescribed the same medications will not meet the inclusion criteria, implying that participants that are enrolled in the trial may not always be a true representation of the population (86, 87).

On the contrary, several recent studies (56, 88–90) have shown use of DOACs to be safe and effective in most obese adults compared to warfarin. These recent findings suggest that the previous threshold of 120 kg may have been conservative and generalized indicating all DOACs have a similar effect. Results from recent studies (61, 78, 91), including the results from this meta-analysis, however, suggest individual superiority of DOACs may exist within the obese adult populations. Further studies are warranted, however, to appreciate the true effect of obesity on DOACs.

## Limitations

This review has several limitations. A key limitation was that we were unable to include the ARISTOTLE and ROCKET-AF trials in our meta-analysis. This meant that we were unable to comment on rivaroxaban and to a certain degree apixaban. Secondly, we did not include non-AF clinical trials and other study designs in our meta-analysis, which may have an impact on the applicability of the results on other conditions, that is, VTE and PE.

## Conclusions

There was no difference between the BMI classes in any of the outcomes assessed. This may be due to the limited number of people in the trial that were in the obese class, especially obese class III. There is an urgent need for large prospective trials with population stratification for the inclusion of obese adults, especially obese class III, to confirm which DOACs are safe and efficacious in these patients and at which dose.

## Author Contributions

FS and CF conceived the study and developed the search strategy, screened, and reviewed articles. FS, CF, and RW wrote and edited the manuscript. RC and SI edited, reviewed the articles, and provided expert opinion. All authors contributed to the article and approved the submitted version.

## Funding

FS was supported by the Australian Government Research Training Program (RTP) through a Western Sydney University Doctoral Scholarship. SI receives funding through a Heart Foundation Future Leader Fellowship by the Heart Foundation (Australia). CF receives funding through a Heart Foundation Postdoctoral Fellowship (Ref: 102168) from the Heart Foundation (Australia) and a NHMRC Emerging Leadership Fellowship (APP 1196262).

## Conflict of Interest

The authors declare that the research was conducted in the absence of any commercial or financial relationships that could be construed as a potential conflict of interest.

## Publisher's Note

All claims expressed in this article are solely those of the authors and do not necessarily represent those of their affiliated organizations, or those of the publisher, the editors and the reviewers. Any product that may be evaluated in this article, or claim that may be made by its manufacturer, is not guaranteed or endorsed by the publisher.
