# Supplementary material for: Effectiveness of Direct Oral Anticoagulants in Obese Adults With Atrial Fibrillation: A Systematic Review of Systematic Reviews and Meta-Analysis
Source: Front Cardiovasc Med. 2021 Oct 8;8:732828. doi: 10.3389/fcvm.2021.732828 (PMC8531486; doi:10.3389/fcvm.2021.732828)
Supplement: Supplementary file 1 [file Data_Sheet_1.docx]

Supplementary Material

# Figures:

## BMI ≥ 25 vs BMI <25 - Entire Trial


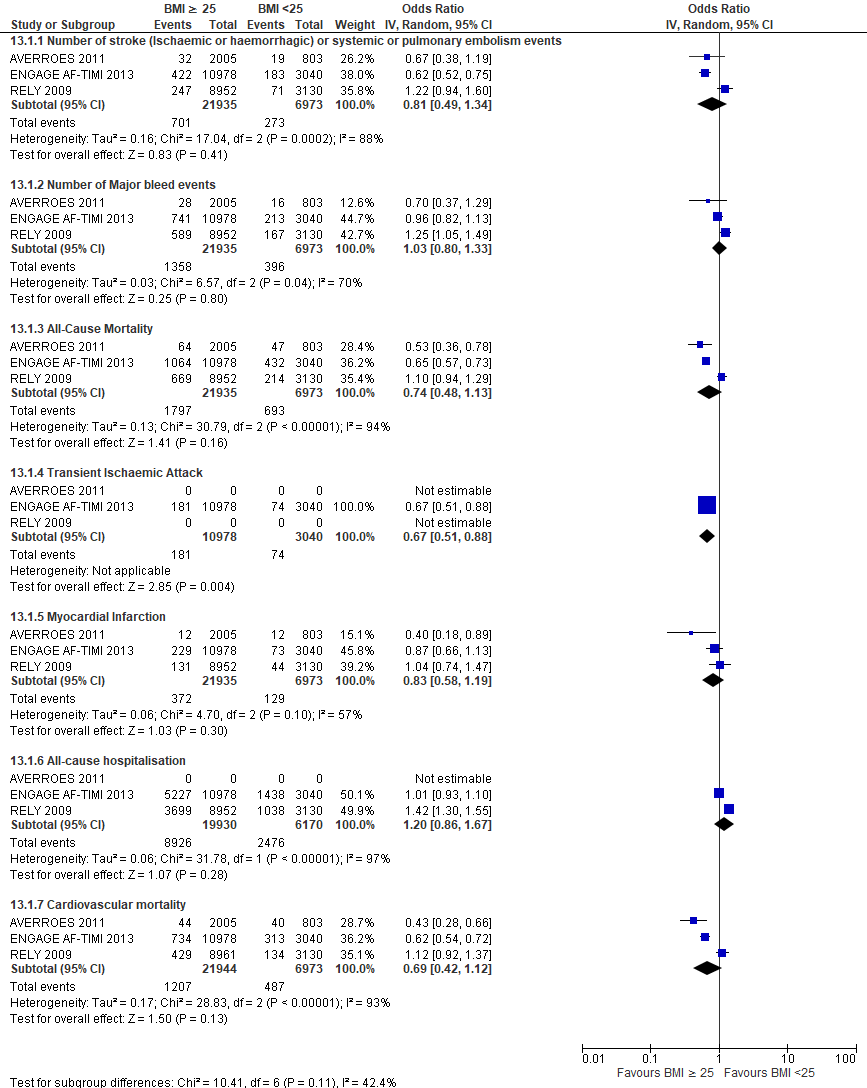


Figure 1: Forest plot of comparison: BMI ≥ 25 vs BMI <25 - Entire Trial

## Normal vs Underweight - Entire Trial


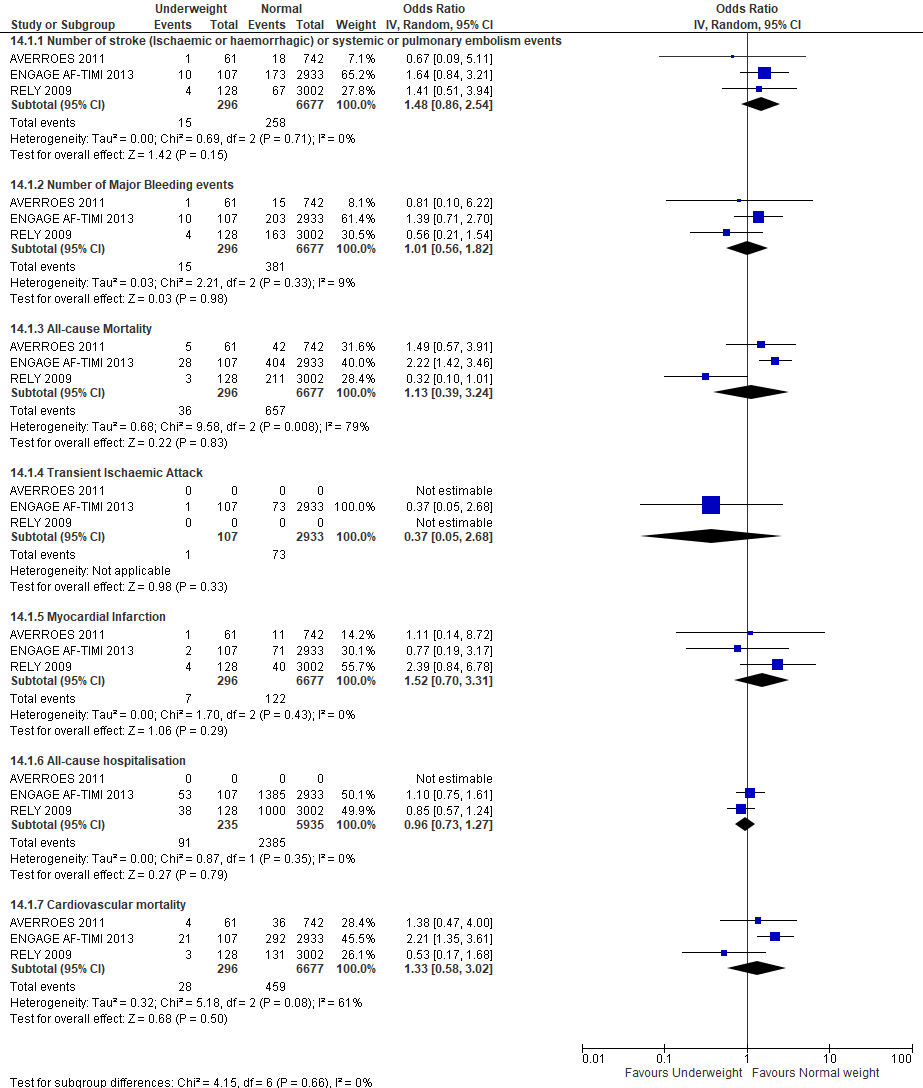


Figure 2: Forest plot of comparison: Normal vs Underweight - Entire Trial

## Normal vs Overweight - Entire Trial


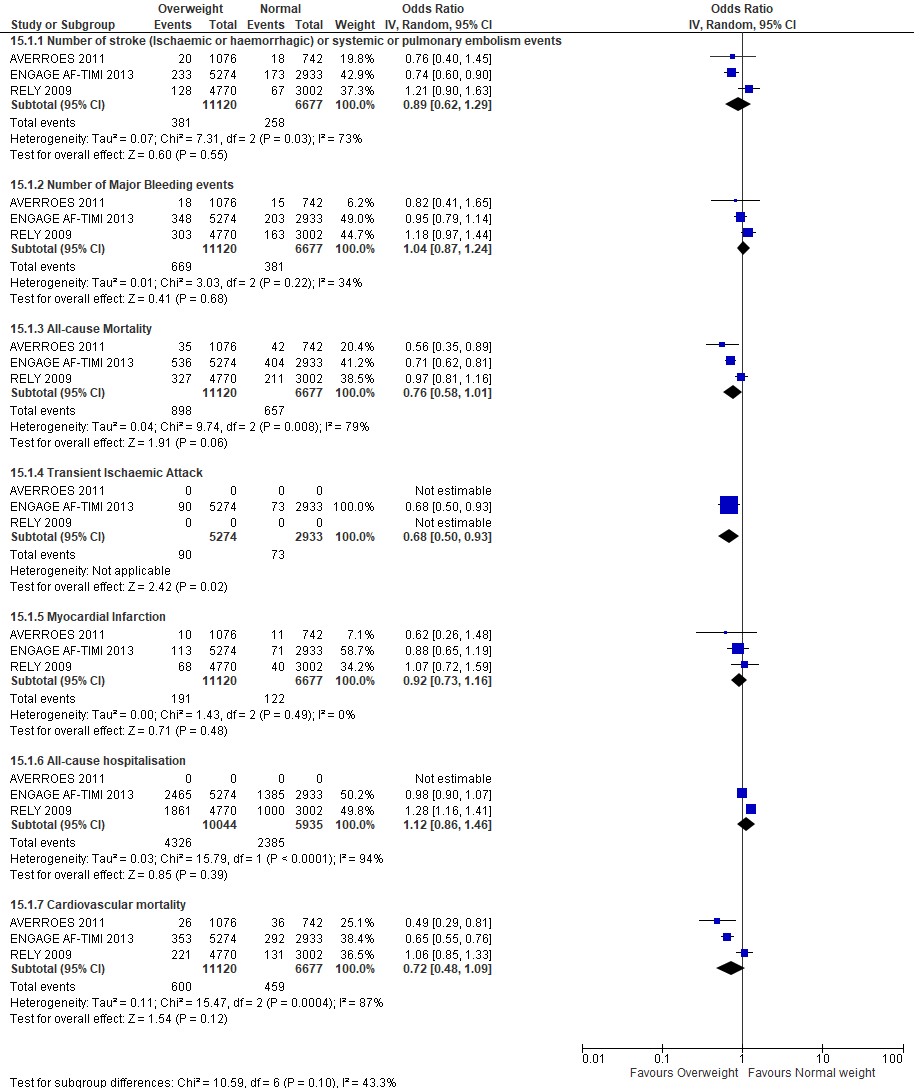


Figure 3: Forest plot of comparison: Normal vs Overweight - Entire Trial

## Normal vs Obese class I - Entire Trial


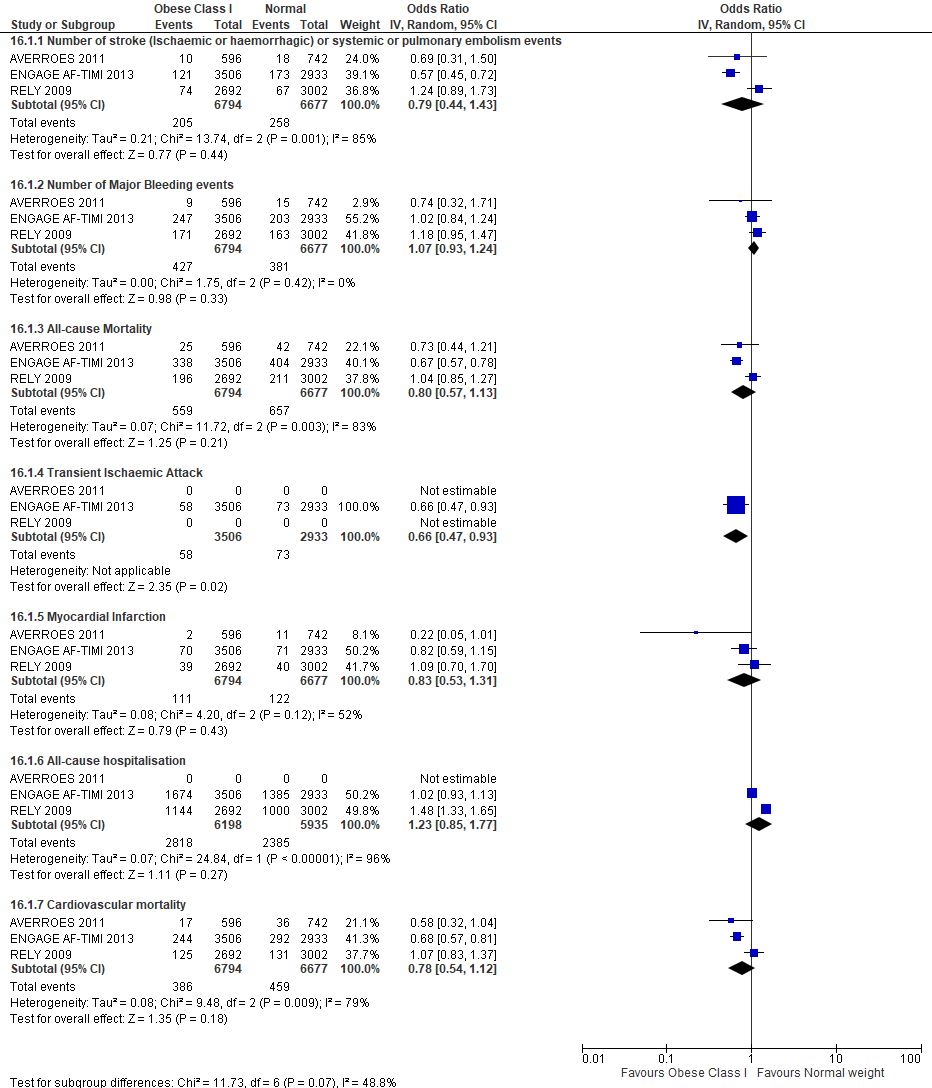


Figure 4: Forest plot of comparison: Normal vs Obese class I - Entire Trial

## Normal vs Obese class II - Entire Trial


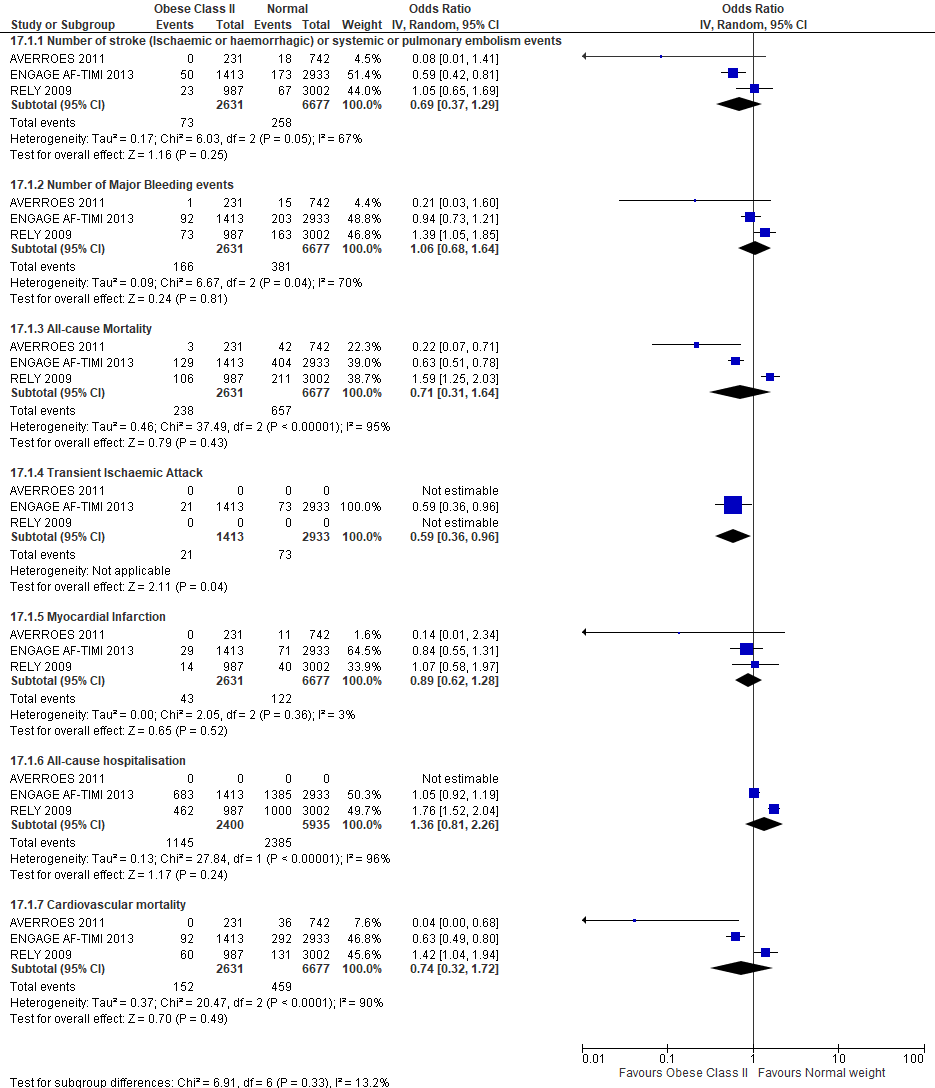


Figure 5: Forest plot of comparison: Normal vs Obese class II - Entire Trial

## Normal vs Obese class III - Entire Trial


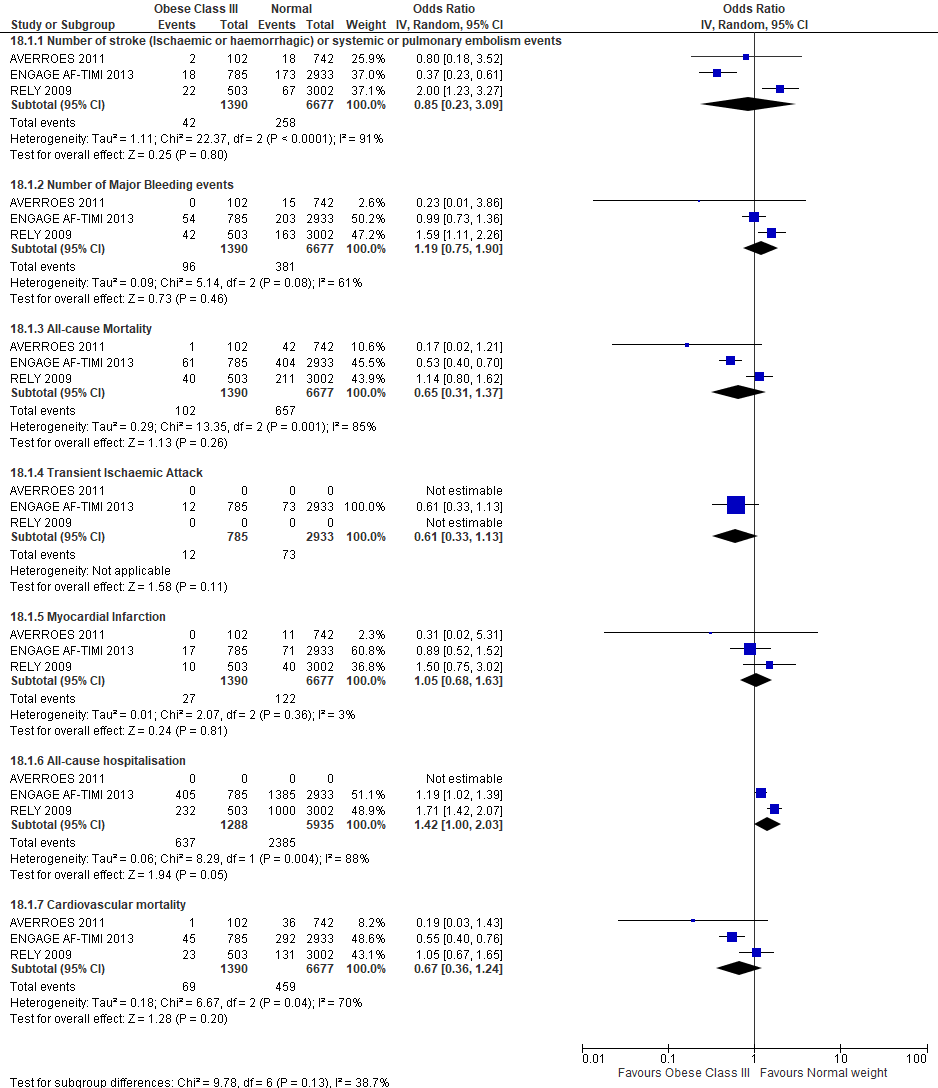


Figure 6: Forest plot of comparison: Normal vs Obese class III - Entire Trial

# Tables:

## Table 1: Excluded Studies from Systematic Review

| Reason for Exclusion | Study Name | Reference |
| --- | --- | --- |
| Different Study Design | Kalani et al 2019 | Kalani C, Awudi E, Alexander T, Udeani G, Surani S. Evaluation of the efficacy of direct oral anticoagulants (DOACs) in comparison to warfarin in morbidly obese patients. Hospital Practice. 2019 Aug 8;47(4):181-5. |
| Conference Proceedings | Fietz et al 2019 | Fietz C, Michels G, Mueller C, Wiesen MH. Monitoring of apixaban in a super obese patient. The American Journal of Medicine. 2019 Jan 1;132(1): e15-16. |
| Letter to editor/commentary | Güler et al 2015 | Güler E, Güler GB, Demir GG, Hatipoğlu S. A review of the fixed dose use of new oral anticoagulants in obese patients: Is it really enough?. Anatolian journal of cardiology. 2015 Dec;15(12):1020-29 |
| Different patient population | Buehler et al 2013 | Buehler KS, Yancey AM. Anticoagulant dosing in obesity should be individualized and drug specific. Drug Topics 2013;157(7) |
| DOACs not reviewed | Martin et al 2016 | Martin K, Beyer-Westendorf J, Davidson BL, Huisman MV, Sandset PM, Moll S. Use of the direct oral anticoagulants in obese patients: guidance from the SSC of the ISTH. Journal of thrombosis and haemostasis: JTH. 2016 Jun;14(6):1308-13 |
| Wrong intervention | Li et al 2016 | Li A, Lopes RD, Garcia DA. Use of direct oral anticoagulants in special populations. Hematology/Oncology Clinics. 2016 Oct 1;30(5):1053-71. |
|  | Badescu et al 2017 | Badescu C, Badulescu O, Nicoleta DI, Badescu L, Rezus C. Oral Anticoagulant Treatment in Obese Patients. The Medical-Surgical Journal. 2017 Jun 30;121(2):308-12. |
|  | Hilleman et al 2019 | Hilleman, D. E., Malesker, M. A., & Hannig, D. Direct-Acting Oral Anticoagulant Use in Special Populations. P&T: A Peer-Reviewed Journal for Managed Care & Formulary Management, 2019 44(12), 738–748. |
|  | Buckley et al 2017 | Buckley LF, Rybak E, Aldemerdash A, Cheng JW, Fanikos J. Direct oral anticoagulants in patients with atrial fibrillation and renal impairment, extremes in weight, or advanced age. Clinical cardiology. 2017 Jan;40(1):46-52. |
|  | González et al 2015 | González Cambeiro MC, Rodríguez Mañero M, Raposeiras Roubin AA, Emad S, González Juanatey JR. Review of obesity and atrial fibrillation: exploring the paradox. Journal of Atrial Fibrillation. 2015 Aug;8(2):21-25 |
|  | De Caterina et al 2017 | De Caterina R, Lip GY. The non-vitamin K antagonist oral anticoagulants (NOACs) and extremes of body weight—a systematic literature review. Clinical Research in Cardiology. 2017 Aug 1;106(8):565-72. |
|  | Kido et al 2020 | Kido K, Lee JC, Hellwig T, Gulseth MP. Use of Direct Oral Anticoagulants in Morbidly Obese Patients. Pharmacotherapy: The Journal of Human Pharmacology and Drug Therapy. 2020 Jan;40(1):72-83. |
|  | Morrill et al 2015 | Morrill AM, Ge D, Willett KC. Dosing of target-specific oral anticoagulants in special populations. Annals of Pharmacotherapy. 2015 Sep;49(9):1031-45. |
| Conference Proceedings | Chugh et al 2018 | Chugh Y, Krishna HB, Ayala RQ, Zepeda I, Li D, Gonzalez CA, Patel K, Gongora C, Kallur R, Rastogi U, Piplani S. Comparative Safety and Efficacy of Rivaroxaban, Dabigatran and Apixaban in Obese and Morbidly Obese Patients with Heart Failure and Non-Valvular Atrial Fibrillation: A Real-World Analysis. Circulation. 2018 Nov 6;138(Suppl_1): A15192 |
|  | Kochawan et al 2016 | Kochawan Boonyawat, Francois Caron, Ang Li, Chatree Chai-Adisaksopha, Wendy Lim, Iorio Alfonso, Renato Delascio Lopes, David A Garcia, Mark Crowther; Association of BW with Thromboembolic and Bleeding Outcomes in Phase III Randomized Controlled Trials of Direct Oral Anticoagulants: Systematic Review and Meta-Analysis. Blood 2016; 128 (22): 88 |
|  | Barakat et al 2019 | Barakat AF, Jain S, Masri A, Senussi M, Wang Y, Bhonsale A, Saba S, Mulukutla SR. Comparative Outcomes of Direct Oral Anticoagulants versus Warfarin in Morbidly Obese Patients with Non-valvular Atrial Fibrillation. Circulation. 2019 Nov 19;140(Suppl_1): A12646 |
|  | Talha et al 2019 | Talha Aijaz, Okechukwu Nwabueze Obi, Nida Khokhar, Prasanth Lingamaneni, Muhammad Zain Farooq; Safety and Efficacy of Direct Oral Anticoagulants in Morbidly Obese Patients: A Meta-Analysis. Blood 2019; 134 (Supplement_1): 1166. |
|  | Bano et al 2017 | Bano S, Salahuddin U, Garg S, Kulkarni N, Anand V, Mayo H, Rao S, Pandey A. Association between Body Mass Index and Outcomes Among Patients with Prevalent Atrial Fibrillation: A Meta-Analysis. Journal of the American College of Cardiology. 2017 Mar 21;69(11S):482 |
|  | Xu et al 2016 | Xu Y, Kus T, Greiss I, Montigny M, Sarrazin JF, Dion D, Breton R, Essebag V, Mardigyan V, Rudski L, Garcia MB. The impact of body weight on adverse outcomes with novel oral anticoagulants and Warfarin: preliminary results from the integrate study. Journal of the American College of Cardiology. 2016 Apr 5;67(13S):798 |
|  | Briasoulis et al 2020 | Briasoulis A, Mentias A, Alvarez P, Vaughan-Sarrazin M. Outcomes of Obese Patients with Atrial Fibrillation (Af) Receiving Direct Oral Anticoagulants. Journal of the American College of Cardiology. 2020 Mar 24;75(11S1):294 |
|  | Joosten et al 2019 | Joosten, L.; Van Maanen, R.; Van Den Dries, C.; Rutten, F. H.; Hoes, A. W.; Geersing, G. J.; Van Doorn, S. PB0001 Off- label dose reduction of direct oral anticoagulants in atrial fibrillation and venous thromboembolism: A systematic Review and Meta- analysis. Research and Practice in Thrombosis and Haemostasis, 2019 3:(S1):1. Poster presented at the 27th Congress of the International Society on Thrombosis and Haemostasis. Australia. |
|  | Bianco et al 2020 | Bianco C, Wen S, Cai Y, Finch C, Kimble W, Caccamo M, Sokos GG. Novel Oral Anticoagulants Are Safe and Effective for Thromboembolic Prophylaxis in Obese Patients with Atrial Fibrillation. Journal of the American College of Cardiology. 2020 Mar 24;75(11S1):505 |
| Letter to editor/Commentary | Undas 2016 | Undas A. Practical considerations on non-vitamin K oral anticoagulants in patients with high body weight. The Anatolian Journal of Cardiology. 2016 Mar 1;16(3):220 |
|  | Lip et al 2019 | Lip GY, Khan AA, Olshansky B. Short-Term Outcomes of Apixaban Versus Warfarin in Patients with Atrial Fibrillation: Is Body Weight an Important Consideration? 2019 May 139(20):2301-2303 |
|  | Berg 2011 | Berg AM. Dabigatran compared with warfarin for stroke prevention in atrial fibrillation. Annals of internal medicine. 2011 Apr 19;154(8):570. |
|  | Lüscher 2019 | Lüscher TF. Unresolved issues of anticoagulation in atrial fibrillation: age, BMI, reduced dose, and ethnicity. European Heart Journal 2019;40(19):1477-1481 |
|  | den Exter et al 2019 | den Exter PL, Huisman MV. Direct oral anticoagulants and obesity: one size fits all? The Lancet Haematology. 2019 Jul 1;6(7): e341-2. |
|  | Badheka et al 2011 | Badheka AO, Rathod A, Bharadwaj A, Afonso L, Jacob S. Obesity paradox in outcomes of atrial fibrillation. American Journal of Cardiology. 2011 Aug 1;108(3):474. |
|  | Moll et all 2019 | Moll S, Crona DJ, Martin K. Direct oral anticoagulants in extremely obese patients: OK to use?. Research and Practice in Thrombosis and Haemostasis. 2019 Apr;3(2):152-5 |
| Different patient population | Chen et al 2015 | Chen J, Zhuang X, Long M, Su C, Wang L. Efficacy and safety of edoxaban in nonvalvular atrial fibrillation: a meta-analysis of randomized controlled trials. Journal of Stroke and Cerebrovascular Diseases. 2015 Dec 1;24(12):2710-9. |
|  | Briere et al 2019 | Briere JB, Wu O, Bowrin K, Millier A, Toumi M, Taieb V, Levy P, Coleman CI. Impact of methodological choices on a meta-analysis of real-world evidence comparing non-vitamin-K antagonist oral anticoagulants with vitamin K antagonists for the treatment of patients with non-valvular atrial fibrillation. Current medical research and opinion. 2019 Nov 2;35(11):1867-72. |
|  | Cheng et al 2014 | Cheng JW, Barillari G. Non‐vitamin K antagonist oral anticoagulants in cardiovascular disease management: evidence and unanswered questions. Journal of clinical pharmacy and therapeutics. 2014 Apr;39(2):118-35. |
|  | Pirlog et al 2019 | Pirlog AM, Pirlog CD, Maghiar MA. DOACs vs Vitamin K Antagonists: A Comparison of Phase III Clinical Trials and a Prescriber Support Tool. Open access Macedonian journal of medical sciences. 2019 Apr 15;7(7):1226-32 |
| DOACs not reviewed | Wanahita et al 2008 | Wanahita N, Messerli FH, Bangalore S, Gami AS, Somers VK, Steinberg JS. Atrial fibrillation and obesity—results of a meta-analysis. American heart journal. 2008 Feb 1;155(2):310-5 |
|  | Di Minno et al 2017 | Di Minno MN, Ambrosino P, Dentali F. Safety of warfarin in “high-risk” populations: a meta-analysis of randomized and controlled trials. Thrombosis Research. 2017 Feb 1; 150:1-7 |
|  | Zhu et al 2016 | Zhu W, Wan R, Liu F, Hu J, Huang L, Li J, Hong K. Relation of body mass index with adverse outcomes among patients with atrial fibrillation: a meta‐analysis and systematic review. Journal of the American Heart Association. 2016 Sep 9;5(9): e004006. |
| Wrong intervention | Wengen et al 2016 | Wengen Z, Wei L, Linjuan G, Xiaohui Y, Kui H. GW27-e1150 Relation of body mass index with adverse outcomes among patients with atrial fibrillation: a meta-analysis and systematic review. Journal of the American College of Cardiology. 2016 Oct 18;68(16S):C115 |

## Table 2: Excluded studies from meta-analysis

| Exclusion Reason | Author | Reference |
| --- | --- | --- |
| In-vivo study | Bertaggia‐Calderara et al 2018 | Bertaggia‐Calderara D, Kröll D, Gerschheimer C, Nicolas N, Nett P, Stirnimann G, Alberio L. Effect of rivaroxaban on thrombin generation in vivo. A study in obese patients. International journal of laboratory hematology. 2018 Feb;40(1):e11-4. |
| Observational study | Yanagisawa et al 2015 | Yanagisawa S, Inden Y, Yoshida N, Kato H, Miyoshi-Fujii A, Mizutani Y, Ito T, Kamikubo Y, Kanzaki Y, Hirai M, Murohara T. Body mass index is associated with prognosis in Japanese elderly patients with atrial fibrillation: an observational study from the outpatient clinic. Heart and vessels. 2016 Sep 1;31(9):1553-61. |
| Post hoc analysis of observational data | Inoue et al 2016 | Inoue H, Kodani E, Atarashi H, Okumura K, Yamashita T, Origasa H, J-RHYTHM Registry Investigators. Impact of body mass index on the prognosis of Japanese patients with non-valvular atrial fibrillation. The American journal of cardiology. 2016 Jul 15;118(2):215-21. |
| Post hoc analysis of RCT | Piccini et al 2013 | Piccini JP, Stevens SR, Chang Y, Singer DE, Lokhnygina Y, Go AS, Patel MR, Mahaffey KW, Halperin JL, Breithardt G, Hankey GJ. Renal dysfunction as a predictor of stroke and systemic embolism in patients with nonvalvular atrial fibrillation: validation of the R2CHADS2 Index in the ROCKET AF (Rivaroxaban Once-daily, oral, direct factor Xa inhibition Compared with vitamin K antagonism for prevention of stroke and Embolism Trial in Atrial Fibrillation) and ATRIA (AnTicoagulation and Risk factors In Atrial fibrillation) Study Cohorts. Circulation. 2013 Jan 15;127(2):224-32. |
|  | Ardestani et al 2010 | Ardestani A, Hoffman HJ, Cooper HA. Obesity and outcomes among patients with established atrial fibrillation. Am J Cardiol. 2010 Aug 1;106(3):369-73 |
|  | Badheka et al 2010 | Badheka AO, Rathod A, Kizilbash MA, Garg N, Mohamad T, Afonso L, Jacob S. Influence of obesity on outcomes in atrial fibrillation: yet another obesity paradox. The American journal of medicine. 2010 Jul 1;123(7):646-51. |
|  | Senoo et al 2016 | Senoo K, Lip GY. Body mass index and adverse outcomes in elderly patients with atrial fibrillation: The AMADEUS Trial. Stroke. 2016 Feb;47(2):523-6. |
|  | Proietti et al 2016 | Proietti M, Lane DA, Lip GY. Relation of nonvalvular atrial fibrillation to body mass index (from the SPORTIF Trials). The American journal of cardiology. 2016 Jul 1;118(1):72-8. |
|  | Eikelboom et al 2011 | Eikelboom JW, Wallentin L, Connolly SJ, Ezekowitz M, Healey JS, Oldgren J, Yang S, Alings M, Kaatz S, Hohnloser SH, Diener HC. Risk of bleeding with 2 doses of dabigatran compared with warfarin in older and younger patients with atrial fibrillation: an analysis of the randomized evaluation of long-term anticoagulant therapy (RE-LY) trial. Circulation. 2011 May 31;123(21):2363-72. |
|  | Sandhu et al 2015 | Sandhu RK, Ezekowitz J, Andersson U, Alexander J, Granger C, Halvorsen S, Hanna M, Hijazi Z, Jansky P, Lopes R, Wallentin L. Body mass index and outcomes with apixaban versus warfarin in patients with atrial fibrillation in the aristotle (apixaban for reduction in stroke and other thromboembolic events in atrial fibrillation) trial. Journal of the American College of Cardiology. 2015 Mar 17;65(10S): A284 |
|  | Boriani et al 2019 | Boriani G, Ruff CT, Kuder JF, Shi M, Lanz HJ, Rutman H, Mercuri MF, Antman EM, Braunwald E, Giugliano RP. Relationship between body mass index and outcomes in patients with atrial fibrillation treated with edoxaban or warfarin in the ENGAGE AF-TIMI 48 trial. European heart journal. 2019 May 14;40(19):1541-50. |
|  | Balla et al 2017 | Balla SR, Cyr DD, Lokhnygina Y, Becker RC, Berkowitz SD, Breithardt G, Fox KA, Hacke W, Halperin JL, Hankey GJ, Mahaffey KW. Relation of risk of stroke in patients with atrial fibrillation to body mass index (from patients treated with rivaroxaban and warfarin in the rivaroxaban once daily oral direct factor Xa inhibition compared with vitamin K antagonism for prevention of stroke and embolism trial in atrial fibrillation trial). The American Journal of Cardiology. 2017 Jun 15;119(12):1989-96. |
|  | Sandhu et al 2016 | Sandhu RK, Ezekowitz J, Andersson U, Alexander JH, Granger CB, Halvorsen S, Hanna M, Hijazi Z, Jansky P, Lopes RD, Wallentin L. The ‘obesity paradox’in atrial fibrillation: observations from the ARISTOTLE (Apixaban for Reduction in Stroke and Other Thromboembolic Events in Atrial Fibrillation) trial. European Heart Journal. 2016 Oct 7;37(38):2869-78. |
|  | Hohnloser et al 2019 | Hohnloser SH, Fudim M, Alexander JH, Wojdyla DM, Ezekowitz JA, Hanna M, Atar D, Hijazi Z, Bahit MC, Al-Khatib SM, Lopez-Sendon JL. Efficacy and safety of apixaban versus warfarin in patients with atrial fibrillation and extremes in body weight: insights from the ARISTOTLE trial. Circulation. 2019 May 14;139(20):2292-300. |
| Prospective cohort | Overvad et al 2013 | Overvad TF, Rasmussen LH, Skjøth F, Overvad K, Lip GY, Larsen TB. Body mass index and adverse events in patients with incident atrial fibrillation. The American Journal of Medicine. 2013 Jul 1;126(7): 640.e9-17. |
|  | Wang et al 2014 | Wang J, Yang YM, Zhu J, Zhang H, Shao XH, Tian L, Huang B, Yu LT, Gao X, Wang M. Overweight is associated with improved survival and outcomes in patients with atrial fibrillation. Clinical Research in Cardiology. 2014 Jul 1;103(7):533-42. |
|  | Bunch et al 2016 | Bunch TJ, May HT, Bair TL, Crandall BG, Cutler MJ, Jacobs V, Mallender C, Muhlestein JB, Osborn JS, Weiss JP, Day JD. Long-term influence of body mass index on cardiovascular events after atrial fibrillation ablation. Journal of Interventional Cardiac Electrophysiology. 2016 Sep 1;46(3):259-65. |
|  | Barsam et al 2017 | Barsam SJ, Patel JP, Roberts LN, Kavarthapu V, Patel RK, Green B, Arya R. The impact of body weight on rivaroxaban pharmacokinetics. Research and practice in thrombosis and haemostasist. 2017 Oct 9;1(2):180-187. |
| RCT | Granger et al 2011 (Data unavailable for analysis) | Granger CB, Alexander JH, McMurray JJ, Lopes RD, Hylek EM, Hanna M, Al-Khalidi HR, Ansell J, Atar D, Avezum A, Bahit MC, Diaz R, Easton JD, Ezekowitz JA, Flaker G, Garcia D, Geraldes M, Gersh BJ, Golitsyn S, Goto S, Hermosillo AG, Hohnloser SH, Horowitz J, Mohan P, Jansky P, Lewis BS, Lopez-Sendon JL, Pais P, Parkhomenko A, Verheugt FW, Zhu J, Wallentin L; ARISTOTLE Committees and Investigators. Apixaban versus warfarin in patients with atrial fibrillation. The New England Journal of Medicine. 2011 Sep 15;365(11):981-92. |
|  | Patel et al 2011 (Data unavailable for analysis) | Patel MR, Mahaffey KW, Garg J, Pan G, Singer DE, Hacke W, Breithardt G, Halperin JL, Hankey GJ, Piccini JP, Becker RC, Nessel CC, Paolini JF, Berkowitz SD, Fox KA, Califf RM, ROCKET AF Investigators. Rivaroxaban versus warfarin in nonvalvular atrial fibrillation. The New England Journal of Medicine. 2011 Sep 8;365(10):883-91 |
|  | Schulman et al 2009 (Different Population) | Schulman S, Kearon C, Kakkar AK, Mismetti P, Schellong S, Eriksson H, Baanstra D, Schnee J, Goldhaber SZ; RE-COVER Study Group. Dabigatran versus warfarin in the treatment of acute venous thromboembolism. The New England Journal of Medicine. 2009 Dec 10;361(24):2342-52. |
|  | Schulman et al 2014 (Different Population) | Schulman S, Kakkar AK, Goldhaber SZ, Schellong S, Eriksson H, Mismetti P, Christiansen AV, Friedman J, Le Maulf F, Peter N, Kearon C; RE-COVER II Trial Investigators. Treatment of acute venous thromboembolism with dabigatran or warfarin and pooled analysis. Circulation. 2014 Feb 18;129(7):764-72. |
|  | Bauersachs et al 2010 (Different Population) | EINSTEIN Investigators, Bauersachs R, Berkowitz SD, Brenner B, Buller HR, Decousus H, Gallus AS, Lensing AW, Misselwitz F, Prins MH, Raskob GE, Segers A, Verhamme P, Wells P, Agnelli G, Bounameaux H, Cohen A, Davidson BL, Piovella F, Schellong S. Oral rivaroxaban for symptomatic venous thromboembolism. The New England Journal of Medicine. 2010 Dec 23;363(26):2499-510 |
|  | Buller et al 2012 (Different Population) | EINSTEIN–PE Investigators, Büller HR, Prins MH, Lensin AW, Decousus H, Jacobson BF, Minar E, Chlumsky J, Verhamme P, Wells P, Agnelli G, Cohen A, Berkowitz SD, Bounameaux H, Davidson BL, Misselwitz F, Gallus AS, Raskob GE, Schellong S, Segers A. Oral rivaroxaban for the treatment of symptomatic pulmonary embolism. The New England Journal of Medicine. 2012 Apr 5;366(14):1287-97 |
|  | Agnelli et al 2013 (Different Population) | Agnelli G, Buller HR, Cohen A, Curto M, Gallus AS, Johnson M, Masiukiewicz U, Pak R, Thompson J, Raskob GE, Weitz JI; AMPLIFY Investigators. Oral apixaban for the treatment of acute venous thromboembolism. The New England Journal of Medicine. 2013 Aug 29;369(9):799-808. |
|  | Buller et al 2013 (Different Population) | Hokusai-VTE Investigators, Büller HR, Décousus H, Grosso MA, Mercuri M, Middeldorp S, Prins MH, Raskob GE, Schellong SM, Schwocho L, Segers A, Shi M, Verhamme P, Wells P. Edoxaban versus warfarin for the treatment of symptomatic venous thromboembolism. The New England Journal of Medicine. 2013 Oct 10;369(15):1406-15. |
|  | Upreti et al 2013 (Different Population) | Upreti VV, Wang J, Barrett YC, Byon W, Boyd RA, Pursley J, LaCreta FP, Frost CE. Effect of extremes of body weight on the pharmacokinetics, pharmacodynamics, safety and tolerability of apixaban in healthy subjects. Br J Clin Pharmacol. 2013 Dec;76(6):908-16. |
|  | Kubitza et al 2007 (Different Population) | Kubitza D, Becka M, Zuehlsdorf M, Mueck W. Body weight has limited influence on the safety, tolerability, pharmacokinetics, or pharmacodynamics of rivaroxaban (BAY 59-7939) in healthy subjects. J Clin Pharmacol. 2007 Feb;47(2):218-26. |
| Retrospective cohort | Wang et al 2015 | Wang HJ, Si QJ, Shan ZL, Guo YT, Lin K, Zhao XN, Wang YT. Effects of body mass index on risks for ischemic stroke, thromboembolism, and mortality in Chinese atrial fibrillation patients: a single-center experience. PLoS One. 2015 Apr 7;10(4): e0123516. |
|  | Park et al 2017 | Park CS, Choi EK, Kim HM, Lee SR, Cha MJ, Oh S. Increased risk of major bleeding in underweight patients with atrial fibrillation who were prescribed non-vitamin K antagonist oral anticoagulants. Heart Rhythm. 2017 Apr 1;14(4):501-7. |
|  | Lee et al 2019 | Lee SR, Choi EK, Park CS, Han KD, Jung JH, Oh S, Lip GY. Direct oral anticoagulants in patients with nonvalvular atrial fibrillation and low body weight. Journal of the American College of Cardiology. 2019 Mar 5;73(8):919-31. |
|  | Kwon et al 2016 | Kwon Y, Norby FL, Jensen PN, Agarwal SK, Soliman EZ, Lip GY, Longstreth Jr WT, Alonso A, Heckbert SR, Chen LY. Association of smoking, alcohol, and obesity with cardiovascular death and ischemic stroke in atrial fibrillation: The Atherosclerosis Risk in Communities (ARIC) Study and Cardiovascular Health Study (CHS). PLoS One. 2016 Jan 12;11(1): e0147065. |
|  | Pandey et al 2016 | Pandey A, Gersh BJ, McGuire DK, Shrader P, Thomas L, Kowey PR, Mahaffey KW, Hylek E, Sun S, Burton P, Piccini J. Association of body mass index with care and outcomes in patients with atrial fibrillation: results from the ORBIT-AF registry. JACC: Clinical Electrophysiology. 2016 Jun 1;2(3):355-63. |
|  | Perales et al 2019 | Perales IJ, San Agustin K, DeAngelo J, Campbell AM. Rivaroxaban versus warfarin for stroke prevention and venous thromboembolism treatment in extreme obesity and high body weight. Annals of Pharmacotherapy. 2020 Apr;54(4):344-50. |
|  | Piran et al 2018 | Piran, S., Traquair, H., Chan, N., Bhagirath, V., & Schulman, S. (2018). Peak plasma concentration of direct oral anticoagulants in obese patients weighing over 120 kilograms: A retrospective study. Research and practice in thrombosis and haemostasis, 2(4), 684–688. |
|  | Peterson et al 2019 | Peterson ED, Ashton V, Chen YW, Wu B, Spyropoulos AC. Comparative effectiveness, safety, and costs of rivaroxaban and warfarin among morbidly obese patients with atrial fibrillation. American heart journal. 2019 Jun 1; 212:113-9. |
|  | Kushnir et al 2019 | Kushnir M, Choi Y, Eisenberg R, Rao D, Tolu S, Gao J, Mowrey W, Billett HH. Efficacy and safety of direct oral factor Xa inhibitors compared with warfarin in patients with morbid obesity: a single-centre, retrospective analysis of chart data. The Lancet Haematology. 2019 Jul 1;6(7): e359-65. |
|  | Kido et al 2019 | Kido K, Ngorsuraches S. Comparing the efficacy and safety of direct oral anticoagulants with warfarin in the morbidly obese population with atrial fibrillation. Annals of Pharmacotherapy. 2019 Feb;53(2):165-70. |
| Systematic review & Meta-analysis | Proietti et al 2017 | Proietti M, Guiducci E, Cheli P, Lip GY. Is There an Obesity Paradox for Outcomes in Atrial Fibrillation? A Systematic Review and Meta-Analysis of Non–Vitamin K Antagonist Oral Anticoagulant Trials. Stroke. 2017 Apr;48(4):857-66. |
| Conference Proceedings | Prins et al 2015 | Prins M, Nisio M, Vedovati M, Riera-Mestre A, Mueller K, Cohen A, Wells P, Beyer-Westendorf J, Brighton T, Bounameaux H, Schneider J. Fixed-dose rivaroxaban is not associated with increased recurrent venous thromboembolism or major bleeding in patients with a high or low body weight: AS099. Journal of Thrombosis and Haemostasis. 2015 Jun; 13:35-6. |
| Unavailable | Eikelboom et al 2009 | Unavailable |

## Table 3: Obese population (%) in Trials

| BMI Groups | | | | | | | | |
| --- | --- | --- | --- | --- | --- | --- | --- | --- |
| Trials | ≥ 25 | <25 | Underweight | Normal weight | Overweight | Obese Class I | Obese Class II | Obese Class III |
| RE-LY | 74.04% | 25.89% | 1.06% | 24.83% | 39.45% | 22.26% | 8.16% | 4.16% |
| AVEROSE | 71.40% | 28.60% | 2.17% | 26.42% | 38.32% | 21.23% | 8.23% | 3.63% |
| ENGAGE AF TIMI 48 | 78.31% | 21.69% | 0.76% | 20.92% | 37.62% | 25.01% | 10.08% | 5.60% |

## Table 4: Summary of differences of DOAC trials at 12 months vs entire trial. OV: overweight, UW: underweight, O1: Obese class 1, O2: Obese class 2, O3: Obese class 3, OA: All obese classes, N: Normal; NUW: Normal group in UW vs N, NOV: Normal group in OV vs N, NO1: Normal group in O1 vs N, NO2: Normal group in O2 vs N, NO3: Normal group in O3 vs N, NOA: Normal group in all obese classes’ vs N

| Outcomes | Time | AVERROES (45) | ENGAGE AF TIMI 48 (46) | RELY (32) |
| --- | --- | --- | --- | --- |
| Stroke | 12 Months | ≥25 | ≥25, OV, OA | No difference |
|  | Entire Trial | No difference | ≥25, OV, OA | NO3 |
| Major Bleed | 12 Months | No difference | No difference | No difference |
|  | Entire Trial | No difference | No difference | NO3 |
| All-Cause Mortality | 12 Months | ≥25, OV, O2 | ≥25, NUW, OV, OA | NO2, NO3 |
|  | Entire Trial | ≥25, OV, O2 | ≥25, NUW, OV, OA | NO2 |
| Myocardial Infarction | 12 Months | No difference | No difference | No difference |
|  | Entire Trial | ≥25 | No difference | No difference |
| All-cause hospitalisation | 12 Months | No difference | No difference | ≤25, NOV, NOA |
|  | Entire Trial | No difference | No difference | ≤25, NOV, NOA |
| Cardiovascular Mortality | 12 Months | ≥25, OV, O1, O2 | ≥25, NUW, OV, OA | ≤25, NO2, NO3 |
|  | Entire Trial | ≥25, OV, O2 | ≥25, NUW, OV, OA | No difference |

## Table 5: Full search strategy and results

| **Database** | **Search Terms** | **Results** | **Date** |
| --- | --- | --- | --- |
| **Scopus** | ( TITLE-ABS-KEY ( apixaban ) OR TITLE-ABS-KEY ( rivaroxaban ) OR TITLE-ABS-KEY ( edoxaban ) OR TITLE-ABS-KEY ( dabigatran ) OR TITLE-ABS-KEY ( doac* ) OR TITLE-ABS-KEY ( noac* ) OR TITLE-ABS-KEY ( "novel oral anticoagulant*" ) OR TITLE-ABS-KEY ( "new oral anticoagulant*" ) OR TITLE-ABS-KEY ( "non‐vitamin K oral anticoagulant*" ) OR TITLE-ABS-KEY ( "direct oral anticoagulant*" ) OR TITLE-ABS-KEY ( "direct thrombin inhibitor*" ) OR TITLE-ABS-KEY ( "factor xa inhibitor*" ) OR TITLE-ABS-KEY ( "target specific oral anticoagulant*" ) OR TITLE-ABS-KEY ( tsoac* ) ) AND ( TITLE-ABS-KEY ( obes* ) OR TITLE-ABS-KEY ( overwei* ) OR TITLE-ABS-KEY ( "over eat*" ) OR TITLE-ABS-KEY ( "over feed*" ) OR TITLE-ABS-KEY ( "over fed*" ) OR TITLE-ABS-KEY ( corpulent ) OR TITLE-ABS-KEY ( "body mass index" ) OR TITLE-ABS-KEY ( bmi ) OR TITLE-ABS-KEY ( "adipose tissue*" ) OR TITLE-ABS-KEY ( adipo* ) OR TITLE-ABS-KEY ( "binge eating disorder" ) OR TITLE-ABS-KEY ( "morbid* obes*" ) OR TITLE-ABS-KEY ( "body weight" ) ) AND ( TITLE-ABS-KEY ( af ) OR TITLE-ABS-KEY ( "Atrial fibrillation" ) OR TITLE-ABS-KEY ( "atrial flutter" ) OR TITLE-ABS-KEY ( "atrial fibrillat*" ) OR TITLE-ABS-KEY ( "auricular* fibrillat*" ) OR TITLE-ABS-KEY ( "atrium fibrillat*" ) OR TITLE-ABS-KEY ( "atrial arrhythmi*" ) ) | 489 | 28/04/20 |
| **Cochrane** | AF OR “Atrial fibrillation” OR “atrial flutter” OR “atrial fibrillat*” OR “auricular* fibrillat*” OR “atrium fibrillat*” OR “atrial arrhythmi*” in Title Abstract Keyword AND obes* OR overwei* OR "over eat*" OR "over feed*" OR "over fed*" OR corpulent OR "body mass index" OR bmi OR "adipose tissue*” OR adipo* OR “binge eating disorder" OR "morbid* obes*" OR “body weight” in Title Abstract Keyword AND apixaban OR rivaroxaban OR edoxaban OR dabigatran OR DOAC* OR NOAC* OR “novel oral anticoagulant*” OR “new oral anticoagulant*” OR “non‐vitamin K oral anticoagulant*” OR “direct oral anticoagulant*” OR “direct thrombin inhibitor*” OR “factor xa inhibitor*” OR “target specific oral anticoagulant*” OR TSOAC* in Title Abstract Keyword - (Word variations have been searched) | 0 | 28/04/20 |
| **Web of Science** | #4) #3 AND #2 AND #1  Indexes=SCI-EXPANDED, SSCI, A&HCI, CPCI-S, CPCI-SSH, BKCI-S, BKCI-SSH, ESCI, CCR-EXPANDED, IC Timespan=All years  # 3) TOPIC: (AF OR “Atrial fibrillation” OR “atrial flutter” OR “atrial fibrillat*” OR “auricular* fibrillat*” OR “atrium fibrillat*” OR “atrial arrhythmi*”)  Indexes=SCI-EXPANDED, SSCI, A&HCI, CPCI-S, CPCI-SSH, BKCI-S, BKCI-SSH, ESCI, CCR-EXPANDED, IC Timespan=All years  # 2) TOPIC: (obes* OR overwei* OR "over eat*" OR "over feed*" OR "over fed*" OR corpulent OR "body mass index" OR bmi OR "adipose tissue*” OR adipo* OR “binge eating disorder" OR "morbid* obes*" OR “body weight”)  Indexes=SCI-EXPANDED, SSCI, A&HCI, CPCI-S, CPCI-SSH, BKCI-S, BKCI-SSH, ESCI, CCR-EXPANDED, IC Timespan=All years  # 1) TOPIC: (apixaban OR rivaroxaban OR edoxaban OR dabigatran OR DOAC* OR NOAC* OR “novel oral anticoagulant*” OR “new oral anticoagulant*” OR “non‐vitamin K oral anticoagulant*” OR “direct oral anticoagulant*” OR “direct thrombin inhibitor*” OR “factor xa inhibitor*” OR “target specific oral anticoagulant*” OR TSOAC*)  Indexes=SCI-EXPANDED, SSCI, A&HCI, CPCI-S, CPCI-SSH, BKCI-S, BKCI-SSH, ESCI, CCR-EXPANDED, IC Timespan=All years | 223 | 28/04/20 |
| **Joanna Briggs Institute (Ovid)** | 1. exp Factor Xa Inhibitors/ or Anticoagulants/ or Atrial Fibrillation/ or apixaban.mp.  2. rivaroxaban.mp. or exp Rivaroxaban/  3. Anticoagulants/ or edoxaban.mp.  4. dabigatran.mp. or exp Dabigatran/  5. Atrial Fibrillation/ or Anticoagulants/ or DOAC*.mp.  6. Atrial Fibrillation/ or Anticoagulants/ or NOAC*.mp.  7. Atrial Fibrillation/ or Anticoagulants/ or "novel oral anticoagulant*".mp. or exp Rivaroxaban/  8. Anticoagulants/ or "new oral anticoagulant*".mp. or Atrial Fibrillation/  9. Rivaroxaban/ or Anticoagulants/ or "non‐vitamin K oral anticoagulant*".mp. or Atrial Fibrillation/  10. Anticoagulants/ or "direct oral anticoagulant*".mp. or Atrial Fibrillation/ or Factor Xa Inhibitors/  11. exp Antithrombins/ or Atrial Fibrillation/ or Anticoagulants/ or exp Dabigatran/ or "direct thrombin inhibitor*".mp.  12. exp Rivaroxaban/ or exp Factor Xa Inhibitors/ or Anticoagulants/ or Atrial Fibrillation/ or "factor xa inhibitor*".mp.  13. Atrial Fibrillation/ or Anticoagulants/ or Factor Xa Inhibitors/ or "target specific oral anticoagulant*".mp.  14. Factor Xa Inhibitors/ or Anticoagulants/ or Atrial Fibrillation/ or TSOAC*.mp. or exp Dabigatran/  15. 1 or 2 or 3 or 4 or 5 or 6 or 7 or 8 or 9 or 10 or 11 or 12 or 13 or 14  16. exp Binge-Eating Disorder/ or exp Obesity/ or obes*.mp. or exp Obesity, Morbid/  17. exp Obesity/ or exp Overweight/ or overwei*.mp. or exp Body Weight/ or exp Body Mass Index/  18. "over eat*".mp. or Eating/  19. "over feed*".mp.  20. "over fed*".mp.  21. exp Obesity/ or corpulent.mp.  22. "body mass index".mp. or exp Body Mass Index/  23. exp Obesity/ or exp Body Mass Index/ or bmi.mp.  24. exp Adipose Tissue/ or exp Adipose Tissue, Brown/ or "adipose tissue*".mp. or exp Obesity/ or exp Adipocytes/  25. exp Adipocytes/ or exp Adipose Tissue/ or adipo*.mp. or exp Obesity/  26. "binge eating disorder".mp. or exp Bulimia/ or exp Obesity/ or exp Binge-Eating Disorder/ or exp Feeding Behavior/ or exp "Feeding and Eating Disorders"/  27. exp Obesity/ or exp Obesity, Morbid/ or "morbid* obes*".mp.  28. "body weight".mp. or exp Body Weight/  29. 16 or 17 or 18 or 19 or 20 or 21 or 22 or 23 or 24 or 25 or 26 or 27 or 28  30. exp Heart Atria/ or exp Atrial Fibrillation/ or AF.mp.  31. "Atrial fibrillation".mp. or exp Atrial Fibrillation/  32. "atrial flutter".mp. or exp Atrial Flutter/  33. exp Atrial Fibrillation/ or "atrial fibrillat*".mp.  34. "auricular* fibrillat*".mp. [mp=title, abstract, original title, name of substance word, subject heading word, floating sub-heading word, keyword heading word, organism supplementary concept word, protocol supplementary concept word, rare disease supplementary concept word, unique identifier, synonyms]  35. "atrium fibrillat*".mp. [mp=title, abstract, original title, name of substance word, subject heading word, floating sub-heading word, keyword heading word, organism supplementary concept word, protocol supplementary concept word, rare disease supplementary concept word, unique identifier, synonyms]  36. "atrial arrhythmi*".mp. [mp=title, abstract, original title, name of substance word, subject heading word, floating sub-heading word, keyword heading word, organism supplementary concept word, protocol supplementary concept word, rare disease supplementary concept word, unique identifier, synonyms]  37. 30 or 31 or 32 or 33 or 34 or 35 or 36  38. 15 and 29 and 37  39. limit 38 to yr="2005 -Current" | 2 | 28/04/20 |
| **Medline (Ovid)** |  | 1592 | 28/04/20 |
| **Embase (Ovid)** |  | 7047 | 28/04/20 |
| **CINAHL (EBSCOhost)** | S40) Limiters – Published, Date: 20050101- 20201231, Expanders – Apply equivalent subjects, Search modes - Boolean/Phrase, Interface – EBSCOhost Research Databases, Search Screen – Advanced Search  S39) S15 AND S29 AND S37  S38) S15 AND S29 AND S37  S37) S30 OR S31 OR S32 OR S33 OR S34 OR S35 OR S36  S36) (MH "Arrhythmia, Atrial+") OR (MH "Atrial Fibrillation") OR (MH "Tachycardia, Atrial") OR (MH "Atrial Flutter") OR (MH "Premature Atrial Contractions") OR "“atrial arrhythmi*”"  S35) "“atrium fibrillat*”"  S34) (MH "Atrial Fibrillation") OR (MH "Atrial Flutter") OR "“auricular* fibrillat*”"  S33) (MH "Atrial Fibrillation") OR (MH "Premature Atrial Contractions") OR (MH "Arrhythmia, Atrial") OR (MH "Tachycardia, Atrial") OR (MH "Atrial Flutter") OR "“atrial fibrillat*”"  S32) (MH "Atrial Flutter") OR (MH "Arrhythmia, Atrial+") OR (MH "Atrial Fibrillation") OR (MH  "Tachycardia, Atrial") OR "“atrial flutter”"  S31) (MH "Atrial Fibrillation") OR (MH "Premature Atrial Contractions") OR (MH "Arrhythmia, Atrial+") OR (MH "Atrial Flutter") OR "“Atrial fibrillation”"  S30) AF  S29) S16 OR S17 OR S18 OR S19 OR S20 OR S21 OR S22 OR S23 OR S24 OR S25 OR S26 OR S27 OR S28  S28) (MH "Body Weight+") OR (MH "Body Weight Changes") OR "“body weight”" OR (MH "Body Surface Area") OR (MH "Body Size") OR (MH "Body Image") OR (MH "Body Composition") OR (MH "Body Constitution") OR (MH "Weight Gain") OR (MH "Body Mass Index") OR (MH "Adipose Tissue Distribution") OR (MH "Adipose Tissue")  S27) (MH "Obesity, Morbid") OR ""morbid* obes*""  S26) (MH "Binge Eating Disorder") OR (MH "Eating Disorders+") OR (MH "Feeding and Eating  Disorders of Childhood") OR "“binge eating disorder""  S25) (MH "Adipocytes") OR (MH "Adipose Tissue+") OR (MH "Adipose Tissue Distribution") OR (MH "Adipose Tissue, Beige")  S24) (MH "Adipose Tissue+") OR (MH "Adipose Tissue, Beige") OR (MH "Adipose Tissue Distribution") OR (MH "Abdominal Fat") OR (MH "Neoplasms, Adipose Tissue") OR ""adipose tissue*”" OR (MH "Adipocytes")  S23) "BMI"  S22) (MH "Body Mass Index") OR ""body mass index"" OR (MH "Body Weights and Measures") OR (MH "Body Weight Changes") OR (MH "Body Weight") OR (MH "Body Size") OR (MH "Body Surface Area") OR (MH "Body Composition") OR (MH "Body Constitution") OR (MH "Adipose Tissue Distribution") OR (MH "Adipose Tissue") OR (MH "Pediatric Obesity") OR (MH  "Pharmacokinetics") OR (MH "Obesity") OR (MH "Attitude to Obesity")  S21) "corpulent"  S20) (MH "Food Addiction") OR ""over fed*"" OR (MH "Obesity")  S19) ""over feed*""  S18) (MH "Eating Disorders Management (Iowa NIC)") OR (MH "Eating Disorders") OR (MH  "Eating") OR (MH "Binge Eating Disorder") OR (MH "Food Addiction") OR (MH "Food Habits") OR (MH "Eating Behavior") OR (MH "Feeding and Eating Disorders of Childhood") OR ""over eat*""  S17) "overwei*"  S16) (MH "Obesity+") OR (MH "Obesity, Morbid") OR "obes*"  S15) S1 OR S2 OR S3 OR S4 OR S5 OR S6 OR S7 OR S8 OR S9 OR S10 OR S11 OR S12 OR S13 OR S14  S14) "TSOAC*"  S13) (MH "Anticoagulants") OR "“target specific oral anticoagulant*”"  S12) "“factor xa inhibitor*”"  S11) "“direct thrombin inhibitor*”"  S10) (MH "Anticoagulants") OR "“direct oral anticoagulant*”"  S9) "“non‐vitamin K oral anticoagulant*”"  S8) (MH "Anticoagulants") OR "“new oral anticoagulant*”"  S7) (MH "Anticoagulants") OR "“novel oral anticoagulant*”"  S6) "NOAC*"  S5) "DOAC*"  S4) (MH "Dabigatran Etexilate")  S3) "edoxaban"  S2) (MH "Rivaroxaban") OR "rivaroxaban"  S1) "apixaban" | 194 | 28/04/20 |

**
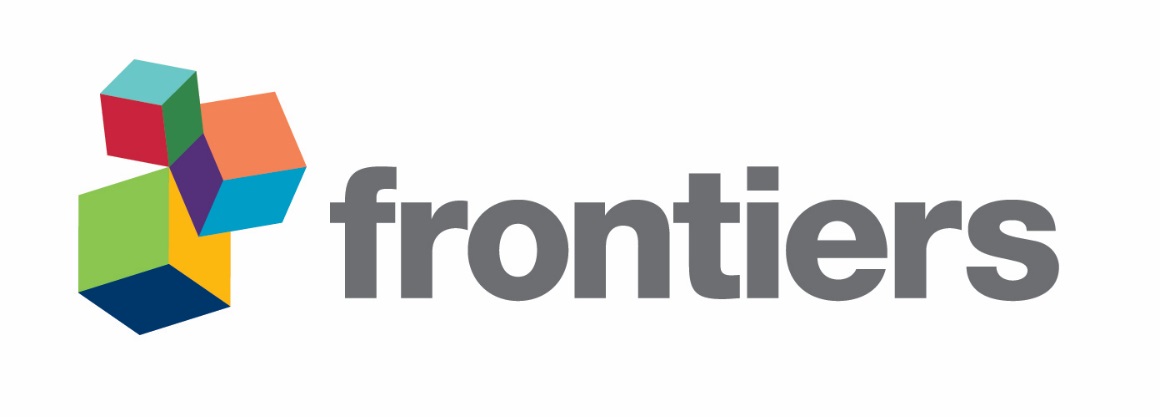
**
